# Supplementary material for: Phylogenetic, Structural and Functional Evolution of the LHC Gene Family in Plant Species
Source: Int J Mol Sci. 2022 Dec 28;24(1):488. doi: 10.3390/ijms24010488 (PMC9820578; doi:10.3390/ijms24010488)
Supplement: Supplementary file 1 [file ijms-24-00488-s001.zip › ijms-2118745-supplementary.pdf]

Table S1. Location information of 1222 LHC genes

Gene_ID	Chr	total length of chromosome or scaffold (bp)	Location (start..end)	Species
GOP47_0029794	21	150368723	121354492..121356043	Adiantum capillus
GOP47_0029810	21	150368723	114709427..114757920	Adiantum capillus
GOP47_0030340	18	157573187	4560728..4608837	Adiantum capillus
GOP47_0029073	18	157573187	4560283..4610444	Adiantum capillus
GOP47_0028116	16	159151851	16549022..16584647	Adiantum capillus
GOP47_0027458	12	161980389	47946386..48006018	Adiantum capillus
GOP47_0024463	8	170047294	124045024..124046186	Adiantum capillus
GOP47_0024603	24	146224257	27925311..27926112	Adiantum capillus
GOP47_0025066	11	163754988	161216288..161217308	Adiantum capillus
GOP47_0023496	22	150505716	93083847..93086587	Adiantum capillus
GOP47_0023994	11	163754988	104907566..104909816	Adiantum capillus
GOP47_0022618	16	159151851	110197002..110284096	Adiantum capillus
GOP47_0022769	3	181400794	142654499..142655680	Adiantum capillus
GOP47_0021906	23	149639048	138155544..138156508	Adiantum capillus
GOP47_0022281	16	159151851	12207563..12209288	Adiantum capillus
GOP47_0020981	28	139379317	17665163..17667505	Adiantum capillus
GOP47_0018395	7	170764101	40192770..40335416	Adiantum capillus
GOP47_0018815	9	165893482	25030289..25031153	Adiantum capillus
GOP47_0019206	20	154628473	129176114..129177022	Adiantum capillus
GOP47_0017791	9	165893482	18580326..18581193	Adiantum capillus
GOP47_0016282	30	114394066	9019057..9038265	Adiantum capillus
GOP47_0016303	4	178378862	102498180..102499125	Adiantum capillus
GOP47_0016742	23	149639048	33723028..33724289	Adiantum capillus
GOP47_0014290	1	194202920	190137290..190137891	Adiantum capillus
GOP47_0012388	29	136679751	27598146..27599558	Adiantum capillus
GOP47_0011504	5	175538835	62098968..62099542	Adiantum capillus
GOP47_0011825	18	157573187	100054644..100055551	Adiantum capillus
GOP47_0009259	9	165893482	35102027..35102921	Adiantum capillus
GOP47_0009265	30	114394066	11343195..11344367	Adiantum capillus
GOP47_0009284	24	146224257	27925189..>27976753	Adiantum capillus
GOP47_0009317	9	165893482	19135246..19136111	Adiantum capillus
GOP47_0009332	17	158492396	91829330..91830127	Adiantum capillus
GOP47_0009860	14	160835734	9188586..9189142	Adiantum capillus
GOP47_0008646	24	146224257	58932107..58933682	Adiantum capillus
GOP47_0007854	30	114394066	4845596..5237887	Adiantum capillus
GOP47_0006615	1	194202920	137495526..137496841	Adiantum capillus
GOP47_0005019	6	174067070	<173126950..173128071	Adiantum capillus
GOP47_0004081	30	114394066	4931994..4932908	Adiantum capillus
GOP47_0003057	22	150505716	60212885..60214136	Adiantum capillus
GOP47_0000643	9	165893482	19058299..>19168504	Adiantum capillus
GOP47_0000966	9	165893482	37957677..37959182	Adiantum capillus
LOC18422362		9150321	NW_006494904.1(5642358..5646961)	Amborella trichopoda

LOC18422010		9150321	NW_006494904.1(8396366..8399175)	Amborella trichopoda
LOC18423121		9389330	NW_006494916.1(9135120..9136628)	Amborella trichopoda
LOC18423737		4476844	NW_006495044.1(224945..240476)	Amborella trichopoda
LOC18423902		4476844	NW_006495044.1(41577..80592)	Amborella trichopoda
LOC18423902		4476844	NW_006495044.1(41577..80592)	Amborella trichopoda
LOC18426364		3167	NW_006497043.1(2156..2797)	Amborella trichopoda
LOC18427642		3069342	NW_006497417.1(2294331..2296125)	Amborella trichopoda
LOC18427807		2678520	NW_006497421.1(1994423..1998173)	Amborella trichopoda
LOC18429159		11522362	NW_006497648.1 (3122845..3125066)	Amborella trichopoda
LOC18429592		11522362	NW_006497648.1(10203265..10205829)	Amborella trichopoda
LOC18429592		11522362	NW_006497648.1(10203265..10205829)	Amborella trichopoda
LOC110006822		11522362	NW_006497648.1(3127973..3129470)	Amborella trichopoda
LOC18430079		5143656	NW_006497754.1(752053..752894)	Amborella trichopoda
LOC18433941		9414115	NW_006498435.1 (6461486..6462500)	Amborella trichopoda
LOC18433883		9414115	NW_006498435.1(4477536..4487446)	Amborella trichopoda
LOC18433883		9414115	NW_006498435.1(4477536..4487446）	Amborella trichopoda
LOC18435402		7204098	NW_006498608.1(2442669..2444322)	Amborella trichopoda
LOC18436215		8757819	NW_006498806.1(6608448..6611459)	Amborella trichopoda
LOC18436726		7341449	NW_006499159.1(2341826..2344391)	Amborella trichopoda
LOC18437148		1154593	NW_006499296.1(873341..876177)	Amborella trichopoda
LOC18437933		6285685	NW_006499435.1(5491359..5496296)	Amborella trichopoda
LOC18438094		8123900	NW_006499436.1 (3029564..3033141)	Amborella trichopoda
LOC18439699		6709877	NW_006499728.1(1439885..1448083)	Amborella trichopoda
LOC18443751		5613473	NW_006499912.1 (5146555..5158495)	Amborella trichopoda
LOC18446791		455365	NW_006500279.1(231591..232735)	Amborella trichopoda
LOC18447397		4329289	NW_006500340.1 (592163..594710)	Amborella trichopoda
AT1G29910	1	30427671	NC_003070.9 (10472280..10473502	Arabidopsis thaliana
AT1G29930	1	30427671	NC_003070.9 (10477885..10479114)	Arabidopsis thaliana
AT1G34000	1	30427671	NC_003070.9 (12357910..12358966	Arabidopsis thaliana
AT1G44575	1	30427671	NC_003070.9 (16870221..16873427)	Arabidopsis thaliana
AT1G45474	1	30427671	NC_003070.9 (17179250..17180806)	Arabidopsis thaliana
AT1G61520	1	30427671	NC_003070.9 (22699715..22701412)	Arabidopsis thaliana
AT1G61520	1	30427671	NC_003070.9 (22699715..22701412)	Arabidopsis thaliana
AT1G76570	1	30427671	NC_003070.9 (28728998..28730851)	Arabidopsis thaliana
AT1G19150	1	30427671	NC_003070.9 (6612630..6613972)	Arabidopsis thaliana
AT2G30390	2	19698289	NC_003071.7 (12950954..12954148	Arabidopsis thaliana
AT2G34420	2	19698289	NC_003071.7 (14522524..14523568	Arabidopsis thaliana
AT2G34430	2	19698289	NC_003071.7 (14524607..14525918)	Arabidopsis thaliana
AT2G40100	2	19698289	NC_003071.7 (16745628..16747424)	Arabidopsis thaliana
AT2G05070	2	19698289	NC_003071.7 (1799234..1800472	Arabidopsis thaliana
AT2G05100	2	19698289	NC_003071.7 (1822990..1824480	Arabidopsis thaliana
AT2G21970	2	19698289	NC_003071.7 (9356983..9357967	Arabidopsis thaliana
AT3G27690	3	23459830	NC_003074.8 (10255906..10257489)	Arabidopsis thaliana
AT3G27690	3	23459830	NC_003074.8 (10255906..10257489)	Arabidopsis thaliana

AT3G47470	3	23459830	NC_003074.8 (17493343..17495033	Arabidopsis thaliana
AT3G54890	3	23459830	NC_003074.8 (20339504..20341176	Arabidopsis thaliana
AT3G61470	3	23459830	NC_003074.8 (22745514..22747444)	Arabidopsis thaliana
AT3G08940	3	23459830	NC_003074.8 (2717557..2718923)	Arabidopsis thaliana
AT3G22840	3	23459830	NC_003074.8 (8084405..8085669	Arabidopsis thaliana
AT4G34190	4	18585056	NC_003075.7 (16372333..16373736	Arabidopsis thaliana
AT4G10340	4	18585056	NC_003075.7 (6408007..6409849)	Arabidopsis thaliana
AT4G14690	4	18585056	NC_003075.7 (8418242..8419325)	Arabidopsis thaliana
AT4G17600	4	18585056	NC_003075.7 (9803624..9805410)	Arabidopsis thaliana
AT5G47110	5	26975502	NC_003076.8 (19133986..19135414	Arabidopsis thaliana
AT5G01530	5	26975502	NC_003076.8 (208866..210548)	Arabidopsis thaliana
AT5G54270	5	26975502	NC_003076.8 (22038165..22039568)	Arabidopsis thaliana
AT5G02120	5	26975502	NC_003076.8 (419091..419773)	Arabidopsis thaliana
AT5G26030	5	26975502	NC_003076.8 (9096451..9099185)	Arabidopsis thaliana
Bathy13g01310	13	708035	NC_023996.1 (315969..317959)	Bathycoccus prasinos
Bathy14g01850	14	663424	NC_023995.1 (336759..337665	Bathycoccus prasinos
Bathy13g02460	13	708035	NC_023996.1 (543781..545028	Bathycoccus prasinos
Bathy12g03300	12	712459	NC_023997.1 (602266..603598	Bathycoccus prasinos
Bathy10g00210	10	794368	NC_023999.1 (27637..28775	Bathycoccus prasinos
Bathy09g00740	9	895536	NC_024000.1 (132799..134106	Bathycoccus prasinos
Bathy04g04390	4	1037991	NC_024005.1 (869486..874267	Bathycoccus prasinos
Bathy03g04620	3	1091008	NC_024006.1 (866903..867967)	Bathycoccus prasinos
Bathy14g02170	14	663424	NC_023995.1 (392568..393122)	Bathycoccus prasinos
Brara.A02052.1.p	A01	29595527	NC_024795.2 (12195446..12196631	Brassica rapa
Brara.B00018.1.p	A01	29595527	NC_024795.2 (19265015..19266308)	Brassica rapa
Brara.A00402.1.p	A01	29595527	NC_024795.2 (1980214..1981303)	Brassica rapa
Brara.F01095.1.p	A01	29595527	NC_024795.2 (1980214..1981303)	Brassica rapa
Brara.A02698.1.p	A01	29595527	NC_024795.2 (21215202..21216685)	Brassica rapa
Brara.B02273.1.p	A02	31442979	NC_024796.2 (13811692..13814200)	Brassica rapa
Brara.D00100.1.p	A02	31442979	NC_024796.2 (23396148..23397442	Brassica rapa
Brara.C02638.1.p	A03	38154160	NC_024797.2 (13465347..13466867)	Brassica rapa
Brara.C03891.1.p	A03	38154160	NC_024797.2 (20183252..20184685	Brassica rapa
Brara.C04035.1.p	A03	38154160	NC_024797.2 (21055243..21056353	Brassica rapa
Brara.C00057.1.p	A03	38154160	NC_024797.2 (231892..232596)	Brassica rapa
Brara.H02384.1.p	A03	38154160	NC_024797.2 (8434140..8435191	Brassica rapa
Brara.F01756.1.p	A04	21928416	NC_024798.2 (17850257..17851256	Brassica rapa
Brara.D00100.1.p	A04	21928416	NC_024798.2 (741622..743257	Brassica rapa
Brara.E01976.1.p	A05	28493056	NC_024799.2 (18231243..18232713)	Brassica rapa
Brara.E03096.1.p	A05	28493056	NC_024799.2 (25647092..25673627	Brassica rapa
Brara.E03123.1.p	A05	28493056	NC_024799.2 (25779254..25780395	Brassica rapa
Brara.E00573.1.p	A05	28493056	NC_024799.2 (3038004..3039406	Brassica rapa
Brara.H00438.1.p	A05	28493056	NC_024799.2 (5718691..5719674	Brassica rapa
Brara.I01499.1.p	A05	28493056	NC_024799.2 (5720147..5721175)	Brassica rapa
Brara.F01756.1.p	A06	29167992	NC_024800.2 (11205684..11207351)	Brassica rapa

Brara.A02541.1.p	A06	29167992	NC_024800.2 (11205684..11207351)	Brassica rapa
Brara.F03187.1.p	A06	29167992	NC_024800.2 (24987303..24988425	Brassica rapa
Brara.F03569.1.p	A06	29167992	NC_024800.2 (27266473..27267828)	Brassica rapa
Brara.F01095.1.p	A06	29167992	NC_024800.2 (6430980..6432108	Brassica rapa
Brara.H01921.1.p	A07	28928902	NC_024801.2 (10634302..10635313	Brassica rapa
Brara.G01647.1.p	A07	28928902	NC_024801.2 (17768260..17769810	Brassica rapa
Brara.G01921.1.p	A07	28928902	NC_024801.2 (19251256..19252836)	Brassica rapa
Brara.E03123.1.p	A08	22981702	NC_024802.2 (17431143..17432208	Brassica rapa
Brara.B00018.1.p	A08	22981702	NC_024802.2 (19820322..19839752	Brassica rapa
Brara.H02525.1.p	A08	22981702	NC_024802.2 (20384712..20385872)	Brassica rapa
Brara.H00558.1.p	A08	22981702	NC_024802.2 (7238267..7239450)	Brassica rapa
Brara.C01741.1.p	A08	22981702	NC_024802.2 672742..4674059	Brassica rapa
Brara.E00982.1.p	A09	45156810	NC_024803.2 (10024985..10026296	Brassica rapa
Brara.H01921.1.p	A09	45156810	NC_024803.2 (14946441..14947793	Brassica rapa
Brara.I02437.1.p	A09	45156810	NC_024803.2 (19216930..19218606	Brassica rapa
Brara.E01939.1.p	A09	45156810	NC_024803.2 (2050491..2051730)	Brassica rapa
Brara.H00470.1.p	A09	45156810	NC_024803.2 (24239055..24240272)	Brassica rapa
Brara.I04910.1.p	A09	45156810	NC_024803.2 (27829980..27831012)	Brassica rapa
Brara.E03123.1.p	A09	45156810	NC_024803.2 (34898952..34900588	Brassica rapa
Brara.D00100.1.p	A09	45156810	NC_024803.2 (38364467..38368049	Brassica rapa
Brara.B00018.1.p	A09	45156810	NC_024803.2 (39875820..39876899)	Brassica rapa
Brara.H02384.1.p	A09	45156810	NC_024803.2 (41458347..41459525)	Brassica rapa
Brara.I02857.1.p	A10	20725698	NC_024804.2 (10248361..10249535)	Brassica rapa
Brara.J00907.1.p	A10	20725698	NC_024804.2 (5106026..5107573)	Brassica rapa
KC19_N004400	11	16661191	CM026432.1	Ceratodon purpureus
KC19_12G03310	12	16459275	CM026433.1	Ceratodon purpureus
KC19_12G03340	12	16459275	CM026433.1	Ceratodon purpureus
KC19_11G04240	11	16661191	CM026432.1	Ceratodon purpureus
KC19_11G06180	11	16661191	CM026432.1	Ceratodon purpureus
KC19_10G04460	10	17229405	CM026431.1	Ceratodon purpureus
KC19_10G09610	10	17229405	CM026431.1	Ceratodon purpureus
KC19_9G112300	9	17527894	CM026430.1	Ceratodon purpureus
KC19_9G141800	9	17527894	CM026430.1	Ceratodon purpureus
KC19_8G155100	8	17567963	CM026429.1	Ceratodon purpureus
KC19_8G164500	8	17567963	CM026429.1	Ceratodon purpureus
KC19_8G164600	8	17567963	CM026429.1	Ceratodon purpureus
KC19_7G150900	7	17972677	CM026428.1	Ceratodon purpureus
KC19_6G040400	6	18980603	CM026427.1	Ceratodon purpureus
KC19_6G126900	6	18980603	CM026427.1	Ceratodon purpureus
KC19_6G139200	6	18980603	CM026427.1	Ceratodon purpureus
KC19_6G139700	6	18980603	CM026427.1	Ceratodon purpureus
KC19_VG219200	6	18980603	CM026427.1	Ceratodon purpureus
KC19_VG270800	6	18980603	CM026427.1	Ceratodon purpureus
KC19_VG270800	6	18980603	CM026427.1	Ceratodon purpureus

KC19_VG270800	6	18980603	CM026427.1	Ceratodon purpureus
KC19_5G012300	5	19969424	CM026425.1	Ceratodon purpureus
KC19_5G191600	5	19969424	CM026425.1	Ceratodon purpureus
KC19_5G191600	5	19969424	CM026425.1	Ceratodon purpureus
KC19_5G191600	5	19969424	CM026425.1	Ceratodon purpureus
KC19_5G191600	5	19969424	CM026425.1	Ceratodon purpureus
KC19_5G191600	5	19969424	CM026425.1	Ceratodon purpureus
KC19_5G191600	5	19969424	CM026425.1	Ceratodon purpureus
KC19_5G191600	5	19969424	CM026425.1	Ceratodon purpureus
KC19_4G013300	4	22785024	CM026424.1	Ceratodon purpureus
KC19_2G137300	2	26629683	CM026422.1	Ceratodon purpureus
KC19_2G137400	2	26629683	CM026422.1	Ceratodon purpureus
KC19_1G032400	1	29001003	CM026421.1	Ceratodon purpureus
KC19_1G064300	1	29001003	CM026421.1	Ceratodon purpureus
KC19_1G064500	1	29001003	CM026421.1	Ceratodon purpureus
KC19_1G064600	1	29001003	CM026421.1	Ceratodon purpureus
KC19_1G064700	1	29001003	CM026421.1	Ceratodon purpureus
KC19_1G065100	1	29001003	CM026421.1	Ceratodon purpureus
KC19_1G321700	1	29001003	CM026421.1	Ceratodon purpureus
KC19_1G321800	1	29001003	CM026421.1	Ceratodon purpureus
Mapa_010273	3	2461926	CM026423.1	Ceratodon purpureus
Mapa_008095	5	19969424	CM026425.1	Ceratodon purpureus
Mapa_008163	2	26629683	CM026422.1	Ceratodon purpureus
Mapa_007988	2	26629683	CM026422.1	Ceratodon purpureus
Mapa_007989	2	26629683	CM026422.1	Ceratodon purpureus
Mapa_007992	8	17567963	CM026429.1	Ceratodon purpureus
Mapa_007995	8	17567963	CM026429.1	Ceratodon purpureus
Mapa_007461	V	2517113	CM026434.1	Ceratodon purpureus
Mapa_006341	11	16661191	CM026432.1	Ceratodon purpureus
Mapa_005629	11	16661191	CM026432.1	Ceratodon purpureus
Mapa_004408	4	22785024	CM026424.1	Ceratodon purpureus
Mapa_004804	4	22785024	CM026424.1	Ceratodon purpureus
Mapa_002052	12	16459275	CM026433.1	Ceratodon purpureus
Mapa_001056	7	17972677	CM026428.1	Ceratodon purpureus
KP509_1Z323500	6	18980603	CM026427.1	Ceratopteris richardii
KP509_1Z323500	2	12149	MU265091.1	Ceratopteris richardii
KP509_1Z118300	11	164513	MU260698.1	Ceratopteris richardii
KP509_37G010900	12	136467727	CM035442.1	Ceratopteris richardii
KP509_37G010900	12	136467727	CM035442.1	Ceratopteris richardii
KP509_36G021200	36	141885106	CM035441.1	Ceratopteris richardii
KP509_36G021200	36	141885106	CM035441.1	Ceratopteris richardii
KP509_21G022000	21	178572925	CM035426.1	Ceratopteris richardii
KP509_20G033200	20	181686993	CM035425.1	Ceratopteris richardii
KP509_19G039000	19	181962917	CM035424.1	Ceratopteris richardii

KP509_19G039000	19	181962917	CM035424.1	Ceratopteris richardii
KP509_19G039000	19	181962917	CM035424.1	Ceratopteris richardii
KP509_14G060500	14	193532319	CM035419.1	Ceratopteris richardii
KP509_13G004200	13	197278082	CM035418.1	Ceratopteris richardii
CBR_g32244		1129207	scaffold_540(11010..79815)	Chara braunii
CBR_g29877		1923690	BFEA01000311.1(46046..51550)	Chara braunii
CBR_g40841		5534110	BFEA01000027.1(118933..120957)	Chara braunii
CBR_g50349		10462021	BFEA01000003.1(5559386..5598044)	Chara braunii
CBR_g9133		9896	BFEA01002107.1(2685..3500)	Chara braunii
CBR_g19118		1109158	BFEA01000531.1(23906..32760)	Chara braunii
CBR_g19126		2350795	BFEA01000226.1(3786..5380)	Chara braunii
CBR_g19130		2350795	BFEA01000226.1(5382..6434)	Chara braunii
CBR_g19131		2350795	BFEA01000226.1(6436..7642)	Chara braunii
CBR_g19133		2350795	BFEA01000226.1(7646..8943)	Chara braunii
CBR_g19139		2497593	BFEA01000209.1(776..92864)	Chara braunii
CBR_g19140		2497593	BFEA01000209.1(92866..93543)	Chara braunii
CBR_g19141		2497593	BFEA01000209.1(93549..10290)	Chara braunii
CBR_g20151		2497593	BFEA01000209.1(10230..23190)	Chara braunii
CBR_g20154		2497593	BFEA01000209.1(23193..32182)	Chara braunii
CBR_g20155		2497593	BFEA01000209.1(92883..93064)	Chara braunii
CBR_g20157		2497593	BFEA01000209.1(93077..102299)	Chara braunii
CBR_g28825		2497593	BFEA01000209.1(102310..10342)	Chara braunii
CBR_g40031		2961896	BFEA01000152.1(33306..38382)	Chara braunii
CBR_g40448		4210221	BFEA01000060.1(16137..21315)	Chara braunii
CBR_g72716		4602786	BFEA01000047.1(1..8365)	Chara braunii
XP_021759163.1		23816425	NW_018742204.1(22631392..22633766)	Chenopodium quinoa
XP_021753376.1		7633013	NW_018742329.1 (3350082..3351110	Chenopodium quinoa
XP_021759743.1		4981908	NW_018742461.1(3326703..3330334)	Chenopodium quinoa
XP_021764007.1		624801	NW_018742487.1 (241831..243845	Chenopodium quinoa
XP_021765781.1		4160932	NW_018742577.1 (1360654..1370901	Chenopodium quinoa
XP_021768049.1		3910945	NW_018742630.1 (2034442..2036346)	Chenopodium quinoa
XP_021769693.1		4616287	NW_018742733.1 (220616..222287)	Chenopodium quinoa
XP_021770863.1		4616287	NW_018742733.1 (3805048..3807767)	Chenopodium quinoa
XP_021771544.1		4654983	NW_018742770.1(1834971..1838191)_	Chenopodium quinoa
XP_021721441.1		4029910	NW_018742879.1 (2096702..2098670)	Chenopodium quinoa
XP_021726887.1		7817241	NW_018742951.1 (7626781..7627821)	Chenopodium quinoa
XP_021729566.1		7817241	NW_018742951.1(7697308..7703033)	Chenopodium quinoa
XP_021731865.1		3488639	NW_018743021.1 (2470552..2472449	Chenopodium quinoa
XP_021736035.1		3488639	NW_018743021.1(2842513..2844787)	Chenopodium quinoa
XP_021736636.1		2711613	NW_018743074.1 (796747..799229	Chenopodium quinoa
XP_021736737.1		185676	NW_018743159.1 (103758..105567)	Chenopodium quinoa
XP_021737646.1		6313176	NW_018743175.1 (2450184..2452132)	Chenopodium quinoa
XP_021739040.1		6313176	NW_018743175.1(2211513..2213818)	Chenopodium quinoa
XP_021743720.1		3632939	NW_018743204.1(3577247..3578539)	Chenopodium quinoa

XP_021744791.1		11561360	NW_018743252.1 (8743336..8746077)	Chenopodium quinoa
XP_021744811.1		11561360	NW_018743252.1(4032033..4034456)	Chenopodium quinoa
XP_021749934.1		4288064	NW_018743285.1(888001..890026)	Chenopodium quinoa
XP_021750786.1		5845939	NW_018743405.1 (2262120..2264675	Chenopodium quinoa
XP_021722909.1		495475	NW_018743548.1 (134546..137821)	Chenopodium quinoa
XP_021754470.1		5234089	NW_018743697.1 (2210916..2211903	Chenopodium quinoa
XP_021755670.1		1847813	NW_018743828.1 (1281972..1288865)	Chenopodium quinoa
XP_021756712.1		1847813	NW_018743828.1 (1281972..1288865)	Chenopodium quinoa
XP_021758637.1		9716283	NW_018743920.1 (9575910..9576926	Chenopodium quinoa
XP_021758723.1		4284168	NW_018743946.1(3045329..3047172)	Chenopodium quinoa
XP_021761611.1		5467236	NW_018744092.1 (2637148..2638595)	Chenopodium quinoa
XP_021763948.1		2879745	NW_018744143.1 (1294997..1296749)	Chenopodium quinoa
XP_021765757.1		268125	NW_018744202.1 (176847..182674	Chenopodium quinoa
XP_021767142.1		10083965	NW_018744367.1 (3211849..3216230	Chenopodium quinoa
XP_021767792.1		5254604	NW_018744537.1 (3093083..3102026	Chenopodium quinoa
XP_021768069.1		5254604	NW_018744537.1 (3093083..3102026	Chenopodium quinoa
XP_021771044.1		1489797	NW_018744560.1 (96600..98685)	Chenopodium quinoa
XP_021775107.1		401834	NW_018744570.1 (364893..376465	Chenopodium quinoa
XP_021776401.1		401834	NW_018744570.1 (364893..376465	Chenopodium quinoa
XP_021716268.1		401834	NW_018744570.1 (364893..376465	Chenopodium quinoa
XP_021717799.1		6278355	NW_018744626.1(4478889..4480816）	Chenopodium quinoa
XP_021717800.1		7402315	NW_018744693.1 (3136314..3149142	Chenopodium quinoa
XP_021720980.1		3870133	NW_018744718.1 (1188746..1196987	Chenopodium quinoa
XP_021724487.1		3490594	NW_018744821.1 (2107155..2112808)	Chenopodium quinoa
XP_021726019.1		6218968	NW_018744878.1 (2083196..2087884	Chenopodium quinoa
XP_021732191.1		3715982	NW_018745080.1 (2552705..2556000)	Chenopodium quinoa
XP_021732292.1		3863660	NW_018745177.1 (2056558..2057724	Chenopodium quinoa
XP_021732293.1		3863660	NW_018745177.1 (2070464..2071732	Chenopodium quinoa
XP_021732295.1		4692610	NW_018745523.1 (146748..151856)	Chenopodium quinoa
XP_021733862.1		763301	NW_018745587.1 (739700..743099)	Chenopodium quinoa
CHLRE_01g016600v5	1	8033585	NC_057004.1（2809303..2811543）	Chlamydomonas reinhardtii
CHLRE_01g016750v5	1	8033585	NC_057004.1（2818212..2820402）	Chlamydomonas reinhardtii
CHLRE_12g508750v5	12	9730733	NC_057015.1（2146536..2149354）	Chlamydomonas reinhardtii
CHLRE_07g339750v5	7	6421821	NC_057010.1（3976768..3981911）	Chlamydomonas reinhardtii
CHLRE_07g344950v5	7	6421821	NC_057010.1（4701800..4704735）	Chlamydomonas reinhardtii
CHLRE_12g548400v5	12	9730733	NC_057015.1（8441637..8443566）	Chlamydomonas reinhardtii
CHLRE_12g548950v5	12	9730733	NC_057015.1（8390064..8391748）	Chlamydomonas reinhardtii

CHLRE_06g283950v5	6	9023763	NC_057009.1（5405936..5407630）	Chlamydomonas reinhardtii
CHLRE_06g284200v5	6	9023763	NC_057009.1（5428650..5433565）	Chlamydomonas reinhardtii
CHLRE_06g284250v5	6	9023763	NC_057009.1（5433613..5437765）	Chlamydomonas reinhardtii
CHLRE_16g673650v5	16	7783580	NC_057019.1（6411057..6413437）	Chlamydomonas reinhardtii
CHLRE_06g272650v5	6	9023763	NC_057009.1（2925547..2927896）	Chlamydomonas reinhardtii
CHLRE_06g251150v5	6	9023763	NC_057009.1（305345..307111）	Chlamydomonas reinhardtii
CHLRE_17g720250v5	17	7188315	NC_057020.1（2974986..2976914）	Chlamydomonas reinhardtii
CHLRE_03g156900v5	3	9219486	NC_057006.1（2108155..2110445）	Chlamydomonas reinhardtii
CHLRE_06g278213v5	6	9023763	NC_057009.1（4069356..4071703）	Chlamydomonas reinhardtii
CHLRE_10g452050v5	10	6576019	NC_057013.1（4509503..4513091）	Chlamydomonas reinhardtii
CHLRE_03g199535v5	3	9219486	NC_057006.1（8435901..8438728）	Chlamydomonas reinhardtii
CHLRE_02g110750v5	2	9223677	NC_057005.1（5786308..5789567）	Chlamydomonas reinhardtii
CHLRE_10g425900v5	10	6576019	NC_057013.1（1162268..1165014）	Chlamydomonas reinhardtii
CHLRE_04g232104v5	4	4091191	NC_057007.1（3996706..3999362）	Chlamydomonas reinhardtii
CHLRE_16g687900v5	16	7783580	NC_057019.1（4587811..4590628）	Chlamydomonas reinhardtii
CHLRE_11g467573v5	11	3826814	NC_057014.1（348422..351813	Chlamydomonas reinhardtii
CHLRE_10g454734v5	10	6576019	NC_057013.1（4900660..4906766）	Chlamydomonas reinhardtii
CHLRE_10g454734v5	10	6576019	NC_057013.1（4900660..4906766）	Chlamydomonas reinhardtii
CHLRE_06g283050v5	6	9023763	NC_057009.1（5311786..5314703）	Chlamydomonas reinhardtii
CHLRE_06g285250v5	6	9023763	NC_057009.1（5548156..5550742）	Chlamydomonas reinhardtii
CHLRE_01g066917v5	1	8033585	NC_057004.1（7954526..7956348）	Chlamydomonas reinhardtii
COCSUDRAFT_19247		1385615	NW_005178025.1(580321..599736)	Coccomyxa subellipsoidea
COCSUDRAFT_37848		1433446	NW_005178027.1(114022..151722)	Coccomyxa subellipsoidea

COCSUDRAFT_64621		3860699	NW_005178041.1(2315499..2329961)	Coccomyxa subellipsoidea
COCSUDRAFT_38026		1404324	NW_005178026.1(1179278..1203472)	Coccomyxa subellipsoidea
COCSUDRAFT_44136		1433446	NW_005178027.1(799742..816976)	Coccomyxa subellipsoidea
COCSUDRAFT_7387		1433446	NW_005178027.1(811979..827731)	Coccomyxa subellipsoidea
COCSUDRAFT_37969		1433446	NW_005178027.1(1376461..1394097)	Coccomyxa subellipsoidea
COCSUDRAFT_25284		1434801	NW_005178028.1(840985..860017)	Coccomyxa subellipsoidea
COCSUDRAFT_25286		1434801	NW_005178028.1(849109..863261)	Coccomyxa subellipsoidea
COCSUDRAFT_48543		1434801	NW_005178028.1(851671..869655)	Coccomyxa subellipsoidea
COCSUDRAFT_30169		1898821	NW_005178031.1(1772215..1791125)	Coccomyxa subellipsoidea
COCSUDRAFT_67011		1898821	NW_005178031.1(1815641..1830918)	Coccomyxa subellipsoidea
COCSUDRAFT_15915		1964347	NW_005178035.1(686323..708894)	Coccomyxa subellipsoidea
COCSUDRAFT_28488		2666267	NW_005178037.1(75594..100886)	Coccomyxa subellipsoidea
COCSUDRAFT_61250		2986206	NW_005178039.1(112590..139909)	Coccomyxa subellipsoidea
COCSUDRAFT_52854		2986206	NW_005178039.1(2048620..2071157)	Coccomyxa subellipsoidea
COCSUDRAFT_58975		3860699	NW_005178041.1(1089741..1109751)	Coccomyxa subellipsoidea
COCSUDRAFT_46127		3860699	NW_005178041.1(1552601..1568168)	Coccomyxa subellipsoidea
COCSUDRAFT_52367		3860699	NW_005178041.1(1552601..1568168)	Coccomyxa subellipsoidea
COCSUDRAFT_35121		3860699	NW_005178041.1(1585631..1604272)	Coccomyxa subellipsoidea
COCSUDRAFT_27246		3860699	NW_005178041.1(2831218..2854545)	Coccomyxa subellipsoidea
COCSUDRAFT_21950		3860699	NW_005178041.1(1681374..1703242)	Coccomyxa subellipsoidea
COCSUDRAFT_64185		4035500	NW_005178042.1(2138956..2140126)	Coccomyxa subellipsoidea
WP_029039165.1		4267208	NZ_CP027107.1(2244855..2251884)	Cronobacter sakazakii
CYME_CMS035C	19	1282939	NC_010145.1 (87011..88294)	Cyanidioschyzon merolae
DUNSADRAFT_5280		436387	MU069638.1(183308..>197622)	Dunaliella salina
DUNSADRAFT_832		559466	MU069544.1(487943..>493894)	Dunaliella salina
DUNSADRAFT_12468		726949	MU069483.1(465064..471496)	Dunaliella salina
DUNSADRAFT_18256		97144	MU070366.1(<7236..>17997)	Dunaliella salina
DUNSADRAFT_16302		140782	MU070180.1(9875..28226)	Dunaliella salina
DUNSADRAFT_10532		296320	MU069823.1(6885..9723)	Dunaliella salina
DUNSADRAFT_5355		434465	MU069641.1(12370..17579)	Dunaliella salina
DUNSADRAFT_3242		497027	MU069587.1(2871..6151)	Dunaliella salina
DUNSADRAFT_1894		534712	MU069560.1(29..2549)	Dunaliella salina
DUNSADRAFT_12648		725702	MU069484.1(5651..12164)	Dunaliella salina
DUNSADRAFT_12651		725702	MU069484.1(15651..16164)	Dunaliella salina
DUNSADRAFT_12471		726949	MU069483.1(20039..33549)	Dunaliella salina
DUNSADRAFT_12475		726949	MU069483.1(20039..33549)	Dunaliella salina
DUNSADRAFT_11311		745454	MU069477.1(42333..>54318)	Dunaliella salina
DUNSADRAFT_3959		956623	MU069452.1(12..1008)	Dunaliella salina
DUNSADRAFT_16877		1125404	MU069443.1(26933..37128)	Dunaliella salina
Esi_0018_0060		1118169	FN648291.1	Ectocarpus siliculosus
WP_067428510.1		1056155	NZ_CP073262.1	Erwinia gerundensis
Eucgr.A01047.1.p	8	74572912	NC_052619.1 (74289329..74296617	Eucalyptus grandis
Eucgr.B03494.1.p	1	53951317	NC_052612.1 (52505488..52506524	Eucalyptus grandis
Eucgr.D00322.1.p	2	58486256	NC_052613.1 (13338188..13339189)	Eucalyptus grandis

Eucgr.D00321.1.p	2	58486256	NC_052613.1 (11686204..11687401	Eucalyptus grandis
Eucgr.F00228.1.p	2	58486256	NC_052613.1 (11682078..11683187	Eucalyptus grandis
Eucgr.H05051.1.p	2	58486256	NC_052613.1 (11677746..11678987	Eucalyptus grandis
Eucgr.J01096.1.p	2	58486256	NC_052613.1 (11669456..11670602	Eucalyptus grandis
Eucgr.K01362.1.p	2	58486256	NC_052613.1 (11688173..11689424	Eucalyptus grandis
Eucgr.K02983.1.p	2	58486256	NC_052613.1 (11700060..11702356	Eucalyptus grandis
Eucgr.A02934.1.p	3	72470685	NC_052614.1 (6809830..6813847)	Eucalyptus grandis
Eucgr.B01008.1.p	5	62829834	NC_052616.1 (4336553..4339199	Eucalyptus grandis
Eucgr.B00523.1.p	6	57658180	NC_052617.1 (54386229..54388561)	Eucalyptus grandis
Eucgr.B01895.2.p	6	57658180	NC_052617.1 (49836483..49838930	Eucalyptus grandis
Eucgr.B01896.1.p	7	60484081	NC_052618.1 (26152204..26154391)	Eucalyptus grandis
Eucgr.B01896.3.p	7	60484081	NC_052618.1 (5765773..5767073)	Eucalyptus grandis
Eucgr.B01008.5.p	7	60484081	NC_052618.1 (29215041..29221717)	Eucalyptus grandis
Eucgr.B01895.3.p	9	44060374	NC_052620.1 (6786973..6789054	Eucalyptus grandis
Eucgr.B01004.1.p	11	50013132	NC_052622.1 (40713541..40714777)	Eucalyptus grandis
Eucgr.C03307.1.p	11	50013132	NC_052622.1 (40713541..40714777)	Eucalyptus grandis
Eucgr.C00637.1.p	1	53951317	NC_052612.1 (34560961..34562840	Eucalyptus grandis
Eucgr.D00319.1.p	2	58486256	NC_052613.1 (53296335..53298214)	Eucalyptus grandis
Eucgr.E00355.1.p	4	39120311	NC_052615.1 (6785164..6786160)	Eucalyptus grandis
Eucgr.L00901.1.p	5	62829834	NC_052616.1 (32742793..32743761	Eucalyptus grandis
Eucgr.F03789.1.p	8	74572912	NC_052619.1 (55600918..55603029)	Eucalyptus grandis
Eucgr.H03224.1.p	10	38819584	NC_052621.1 (12467292..12468808	Eucalyptus grandis
Eucgr.K03092.1.p	11	50013132	NC_052622.1 (18066525..18068239)	Eucalyptus grandis
Eucgr.G01508.1.p	11	50013132	NC_052622.1 (46715371..46716738)	Eucalyptus grandis
Eucgr.I00326.1.p	2	58486256	NC_052613.1 (6712527..6714107	Eucalyptus grandis
Eucgr.K03081.1.p	3	72470685	NC_052614.1 (59550778..59552674	Eucalyptus grandis
Eucgr.K03081.2.p	4	39120311	NC_052615.1 (6772584..6773569	Eucalyptus grandis
WP_010943924.1		3814128	NC_002939.5	Geobacter sulfurreducens
WP_011140841.1		4659019	NC_005125.1	Gloeobacter violaceus
Glyma.10G263000.1.p	1	57932355	NC_016088.4 (40630806..40633299	Glycine max
Glyma.20G129600.2.p	1	57932355	NC_016088.4 (54852651..54854014,	Glycine max
Glyma.07G135300.1.p	1	57932355	NC_016088.4 (56969677..56972077)	Glycine max
Glyma.16G163800.1.p	2	50400358	NC_016089.3 (48295569..48296961	Glycine max
Glyma.01G115900.1.p	2	50400358	NC_016089.4 (49846556..49847566)	Glycine max
Glyma.02G079400.1.p	2	50400358	NC_016089.4 (50118017..50119409	Glycine max
Glyma.04G050400.1.p	2	50400358	NC_016089.4 (5754663..5756927)	Glycine max
Glyma.04G205600.1.p	2	50400358	NC_016089.4 (6822325..6824245)	Glycine max
Glyma.04G249700.1.p	3	46951866	NC_016090.4 (46742139..46743450	Glycine max
Glyma.05G066100.1.p	3	46951866	NC_016090.4 (8807840..8809840)	Glycine max
Glyma.05G183500.1.p	4	51203389	NC_016091.4 (4093975..4097400)	Glycine max
Glyma.05G197600.1.p	4	51203389	NC_016091.4 (40946874..40948736)	Glycine max
Glyma.06G051100.1.p	4	51203389	NC_016091.4 (46638085..46643896)	Glycine max
Glyma.06G159900.1.p	4	51203389	NC_016091.4 (50448855..50450603)	Glycine max
Glyma.07G047600.2.p	5	42274530	NC_038241.2 (32190147..32191209)	Glycine max

Glyma.08G074000.1.p	5	42274530	NC_038241.2 (37102771..37104398)	Glycine max
Glyma.08G141300.1.p	5	42274530	NC_038241.2 (38185994..38192016	Glycine max
Glyma.08G005000.1.p	5	42274530	NC_038241.2 (6601861..6603889)	Glycine max
Glyma.10G177200.1.p	6	50945864	NC_038242.2 (13154385..13160041	Glycine max
Glyma.11G228800.1.p	6	50945864	NC_038242.2 (17361067..17363478	Glycine max
Glyma.15G179400.1.p	6	50945864	NC_038242.2 (2987144..2989168	Glycine max
Glyma.16G016100.1.p	6	50945864	NC_038242.2 (3852438..3856262)	Glycine max
Glyma.16G205200.1.p	7	44949256	NC_038243.2 (15881330..15882542	Glycine max
Glyma.17G147900.1.p	7	44949256	NC_038243.2 (3984022..3986401	Glycine max
Glyma.18G028400.2.p	8	47227184	NC_038244.2 (10881459..10883762)	Glycine max
Glyma.20G127500.1.p	8	47227184	NC_038244.2 (14501718..14503677	Glycine max
Glyma.20G169500.1.p	8	47227184	NC_038244.2 (395223..401131	Glycine max
Glyma.17G262400.2.p	8	47227184	NC_038244.2 (395223..401131	Glycine max
Glyma.16G165800.1.p	8	47227184	NC_038244.2 (5656379..5657717	Glycine max
Glyma.02G305400.1.p	8	47227184	NC_038244.2 (6281654..6282714)	Glycine max
Glyma.03G262300.2.p	9	50572668	NC_038245.2 (38682147..38684905	Glycine max
Glyma.14G008000.1.p	10	51638687	NC_038246.2 (41164108..41166943)	Glycine max
Glyma.13G282000.1.p	10	51638687	NC_038246.2 (45421594..45424173)	Glycine max
Glyma.08G082900.1.p	10	51638687	NC_038246.2 (45421594..45424173)	Glycine max
Glyma.12G219300.1.p	10	51638687	NC_038246.2 (47294609..47296028)	Glycine max
Glyma.10G222100.1.p	10	51638687	NC_038246.2 (48827620..48829650)	Glycine max
Glyma.06G039700.1.p	11	39643745	NC_038247.2 (37282516..37286408	Glycine max
Glyma.08G005000.2.p	12	41531199	NC_038248.2 (39332646..39334503)	Glycine max
Glyma.20G129600.1.p	13	45225048	NC_038249.2 (17570158..17580922)	Glycine max
Glyma.20G127500.2.p	13	45225048	NC_038249.2 (37720927..37722669	Glycine max
Glyma.20G169500.1.p	14	49893278	NC_038250.2 (317763..319492)	Glycine max
Glyma.09G071400.1.p	14	49893278	NC_038250.2 (616572..618276	Glycine max
Glyma.02G309500.1.p	15	53754295	NC_038251.2 (17247678..17250612	Glycine max
Glyma.16G145800.2.p	15	53754295	NC_038251.2 (17247678..17250612	Glycine max
Glyma.03G060300.1.p	15	53754295	NC_038251.2 (4118941..4120385)	Glycine max
Glyma.10G243800.1.p	16	38112070	NC_038252.2 (1395415..1397749	Glycine max
Glyma.20G150600.1.p	16	38112070	NC_038252.2 (30870597..30872862)	Glycine max
Glyma.15G052400.1.p	16	38112070	NC_038252.2 (32472139..32473622)	Glycine max
Glyma.01G204700.1.p	16	38112070	NC_038252.2 (32625795..32626850	Glycine max
Glyma.16G165500.1.p	16	38112070	NC_038252.2 (32640432..32641423	Glycine max
Glyma.08G180000.1.p	16	38112070	NC_038252.2 (32667799..32668742)	Glycine max
Glyma.05G128000.1.p	16	38112070	NC_038252.2 (36833573..36836414	Glycine max
Glyma.16G165200.1.p	17	41740656	NC_038253.2 (12155542..12157603)	Glycine max
Glyma.14G003400.1.p	17	41740656	NC_038253.2 (41686399..41695324)	Glycine max
Glyma.09G154700.2.p	18	58286270	NC_038254.2 (2141177..2144086)	Glycine max
Glyma.02G064700.1.p	20	47846026	NC_038256.2 (36934130..36936853	Glycine max
Glyma.13G078900.1.p	20	47846026	NC_038256.2 (36934130..36936853	Glycine max
Glyma.01G231900.1.p	20	47846026	NC_038256.2 (37060662..37063469)	Glycine max
Glyma.04G167900.4.p	20	47846026	NC_038256.2 (37060662..37063469)	Glycine max

Glyma.06G194900.3.p	20	47846026	NC_038256.2 (38918520..38919980	Glycine max
Glyma.10G222100.2.p	20	47846026	NC_038256.2 (40685773..40689246	Glycine max
Glyma.15G179400.1.p	20	47846026	NC_038256.2 (40685773..40689246	Glycine max
Gohir.D07G011900.1.p	1	55868233	NC_026929.1 (1205865..1206612	Gossypium raimondii
Gohir.D07G015500.1.p	1	55868233	NC_026929.1 (1517925..1520442	Gossypium raimondii
Gohir.D07G049200.1.p	1	55868233	NC_026929.1 (32241809..32243130	Gossypium raimondii
Gohir.D07G070400.1.p	1	55868233	NC_026929.1 (44770640..44771742	Gossypium raimondii
Gohir.D07G171700.1.p	1	55868233	NC_026929.1 (4966008..4967480	Gossypium raimondii
Gohir.D07G197100.1.p	1	55868233	NC_026929.1 (7603639..7604888	Gossypium raimondii
Gohir.D01G056300.1.p	2	62769430	NC_026930.1 (20729217..20730708	Gossypium raimondii
Gohir.D01G107900.1.p	2	62769430	NC_026930.1 (45827901..45830798)	Gossypium raimondii
Gohir.D04G064100.8.p	2	62769430	NC_026930.1 (45827901..45830798)	Gossypium raimondii
Gohir.D04G064100.4.p	2	62769430	NC_026930.1 (45827901..45830798)	Gossypium raimondii
Gohir.D04G064100.8.p	2	62769430	NC_026930.1 (45827901..45830798)	Gossypium raimondii
Gohir.D11G259201.1.p	2	62769430	NC_026930.1 (48350520..48352077	Gossypium raimondii
Gohir.D01G147300.1.p	2	62769430	NC_026930.1 (57704061..57705518	Gossypium raimondii
Gohir.D01G180400.1.p	2	62769430	NC_026930.1 (62433863..62435304)	Gossypium raimondii
Gohir.D01G217600.1.p	2	62769430	NC_026930.1 (9028339..9029526	Gossypium raimondii
Gohir.A02G134801.1.p	3	45765648	NC_026931.1 (13375401..13389835)	Gossypium raimondii
Gohir.D11G232500.2.p	3	45765648	NC_026931.1 (28175424..28180188)	Gossypium raimondii
Gohir.A02G108200.1.p	3	45765648	NC_026931.1 (6307916..6309136)	Gossypium raimondii
Gohir.A08G163300.1.p	4	62178258	NC_026932.1 (50970354..50973247	Gossypium raimondii
Gohir.D08G269600.2.p	4	62178258	NC_026932.1 (61747269..61751197	Gossypium raimondii
Gohir.D02G196000.1.p	5	64140413	NC_026933.1 (60227031..60228193)	Gossypium raimondii
Gohir.D02G207500.1.p	5	64140413	NC_026933.1 (61760898..61761866)	Gossypium raimondii
Gohir.D02G068600.1.p	5	64140413	NC_026933.1 (8885577..8886563)	Gossypium raimondii
Gohir.D09G221400.1.p	6	51074515	NC_026934.1 (48862548..48863455	Gossypium raimondii
Gohir.D09G221500.1.p	6	51074515	NC_026934.1 (48865460..48866473	Gossypium raimondii
Gohir.A11G146500.1.p	7	60982465	NC_026935.1 (14251654..14252978)	Gossypium raimondii
Gohir.D12G152700.1.p	8	57128820	NC_026936.1 (43430896..43432761)	Gossypium raimondii
Gohir.D12G180700.1.p	8	57128820	NC_026936.1 (47802843..47804232)	Gossypium raimondii
Gohir.D12G215500.1.p	8	57128820	NC_026936.1 (51742619..51743859	Gossypium raimondii
Gohir.A04G065200.3.p	9	70713020	NC_026937.1 (12022754..12023917	Gossypium raimondii
Gohir.A04G065200.1.p	9	70713020	NC_026937.1 (19787102..19788662	Gossypium raimondii
Gohir.D05G361900.1.p	9	70713020	NC_026937.1 (21287181..21288599	Gossypium raimondii
Gohir.A05G087400.1.p	9	70713020	NC_026937.1 (21649881..21651443)	Gossypium raimondii
Gohir.A05G150200.1.p	9	70713020	NC_026937.1 (22077311..22080126	Gossypium raimondii
Gohir.A05G248600.1.p	9	70713020	NC_026937.1 (59694002..59694915)	Gossypium raimondii
Gohir.A05G252900.1.p	9	70713020	NC_026937.1 (59694002..59694915)	Gossypium raimondii
Gohir.A05G246000.1.p	9	70713020	NC_026937.1 (6612217..6613589)	Gossypium raimondii
Gohir.D05G237400.1.p	9	70713020	NC_026937.1 (67608997..67610559)	Gossypium raimondii
Gohir.D06G141500.1.p	10	62175169	NC_026938.1 (47754358..47755548)	Gossypium raimondii
Gohir.D06G169100.1.p	10	62175169	NC_026938.1 (55989543..55991435)	Gossypium raimondii
Gohir.D10G039500.1.p	11	62681010	NC_026939.1 (3089394..3090353)	Gossypium raimondii

Gohir.D10G247500.1.p	11	62681010	NC_026939.1 (61813935..61817518	Gossypium raimondii
Gohir.D10G082100.1.p	11	62681010	NC_026939.1 (9314904..9317324	Gossypium raimondii
Gohir.D10G082100.1.p	11	62681010	NC_026939.1 (9314904..9317324	Gossypium raimondii
Gohir.D04G040200.1.p	12	35429946	NC_026940.1 (5665886..5675191)	Gossypium raimondii
Gohir.D13G023200.1.p	13	58321163	NC_026941.1 (1950520..1952203	Gossypium raimondii
WP_113270043.1			Un	Halomonas sulfidaeris
LOC123428101	1H	516505932	NC_058518.1 (175269240..175288394	Hordeum vulgare
LOC123443667	1H	516505932	NC_058518.1 (443808575..443809497)	Hordeum vulgare
LOC123443671	1H	516505932	NC_058518.1 (175269240..175288394	Hordeum vulgare
LOC123410351	1H	516505932	NC_058518.1 (323679939..323680824	Hordeum vulgare
LOC123413393	1H	516505932	NC_058518.1 (443668194..443669107)	Hordeum vulgare
LOC123431105	2H	665585731	NC_058519.1 (639540277..639541692	Hordeum vulgare
LOC123443801	3H	621516506	NC_058520.1 (340751182..340752791	Hordeum vulgare
LOC123444787	3H	621516506	NC_058520.1 (536195417..536198322	Hordeum vulgare
LOC123446703	4H	610333535	NC_058521.1 (503478919..503479809)	Hordeum vulgare
LOC123446768	4H	610333535	NC_058521.1 (526404727..526405659)	Hordeum vulgare
LOC123447843	4H	610333535	NC_058521.1 (52116098..52120227)	Hordeum vulgare
LOC123395457	5H	588218686	NC_058522.1 (379445140..379450658)	Hordeum vulgare
LOC123396572	5H	588218686	NC_058522.1 (439908510..439909290)	Hordeum vulgare
LOC123396573	5H	588218686	NC_058522.1 (439914759..439915594)	Hordeum vulgare
LOC123397537	5H	588218686	NC_058522.1 (439497157..439497915	Hordeum vulgare
LOC123398192	5H	588218686	NC_058522.1 (439655062..439655888)	Hordeum vulgare
LOC123398192	5H	588218686	NC_058522.1 (439655062..439655888)	Hordeum vulgare
LOC123399661	5H	588218686	NC_058522.1 (439587942..439588739	Hordeum vulgare
LOC123399662	5H	588218686	NC_058522.1 (439581987..439582816	Hordeum vulgare
LOC123399663	5H	588218686	NC_058522.1 (439597060..439597897	Hordeum vulgare
LOC123399770	5H	588218686	NC_058522.1 (439789846..439790599)	Hordeum vulgare
LOC123399771	5H	588218686	NC_058522.1 (439808255..439809115)	Hordeum vulgare
LOC123399772	5H	588218686	NC_058522.1 (439814977..439815774)	Hordeum vulgare
LOC123402098	6H	561794515	NC_058523.1 (557732025..557732942)	Hordeum vulgare
LOC123406355	6H	561794515	NC_058523.1 (30949987..30952051)	Hordeum vulgare
MD02G1095600	16	41441581	NC_041804.1 (18095865..18096809	Malus domestica
MD08G1220900	17	34817048	NC_041805.1 (9813593..9814828	Malus domestica
MD03G1218900	17	34817048	NC_041805.1 (10847124..10848837	Malus domestica
MD04G1151300	11	42925075	NC_041799.1 (34408088..34409671)	Malus domestica
MD06G1195000	15	55080361	NC_041803.1 (37095099..37096073	Malus domestica
MD15G1348200	10	41841605	NC_041798.1 (35931969..35932919)	Malus domestica
MD08G1162600	5	48068851	NC_041793.1 (106633..109194)	Malus domestica
MD05G1032800	9	37676754	NC_041797.1 (37638510..37639557)	Malus domestica
MD12G1256700	12	33134071	NC_041800.1 (22537549..22538530	Malus domestica
MD00G1103100	13	44437459	NC_041801.1 (17855830..17857104	Malus domestica
MD15G1083400	13	44437459	NC_041801.1 (17855830..17857104	Malus domestica
MD17G1113400	15	55080361	NC_041803.1 (4141853..4145192	Malus domestica
MD17G1123800	4	32357154	NC_041792.1 (6783239..6816366	Malus domestica

MD11G1236900	5	48068851	NC_041793.1 (106633..109194)	Malus domestica
MD10G1265400	4	32357154	NC_041792.1 (21993707..21994746	Malus domestica
MD05G1000500	6	37231166	NC_041794.1 (27918978..27920156)	Malus domestica
MD09G1292900	5	48068851	NC_041793.1 (106633..109194)	Malus domestica
MD12G1145500	3	37690471	NC_041791.1 (30498972..30500631)	Malus domestica
MD06G1134100	15	55080361	NC_041803.1 (10106414..10107739	Malus domestica
MD09G1134800	4	32357154	NC_041792.1 (24028975..24030628	Malus domestica
MD13G1119100	4	32357154	NC_041792.1 (30853288..30858120)	Malus domestica
MD09G1292900	5	48068851	NC_041793.1 (106633..109194)	Malus domestica
MD00G1103100	6	37231166	NC_041794.1 (6434900..6436870	Malus domestica
MD01G1132800	5	48068851	NC_041793.1 (106633..109194)	Malus domestica
MD01G1174500	6	37231166	NC_041794.1 (33038268..33039619	Malus domestica
MD04G1055800	1	32709648	NC_041789.1 (27626175..27627869)	Malus domestica
MD04G1132800	15	55080361	NC_041803.1 (41344379..41347131)	Malus domestica
MD04G1239600	8	31666303	NC_041796.1 (19135627..19138393)	Malus domestica
MD05G1289300	5	48068851	NC_041793.1 (5403034..5404540	Malus domestica
MD05G1087900	12	33134071	NC_041800.1 (32507966..32511945	Malus domestica
MD06G1047300	2	37631755	NC_041790.1 (7558074..7559705	Malus domestica
MD08G1044400	13	44437459	NC_041801.1 (8751201..8753113)	Malus domestica
MD10G1033800	13	44437459	NC_041801.1 (17862135..17863456	Malus domestica
MD13G1200600	8	31666303	NC_041796.1 (28416616..28417729	Malus domestica
MD13G1200300	14	32560231	NC_024252.1 (2370351..2372293)	Malus domestica
MD13G1200100	15	55080361	NC_041803.1 (5705830..5707694)	Malus domestica
MD13G1200100	13	44437459	NC_041801.1 (17883839..17884735	Malus domestica
MD14G1150400	13	44437459	NC_041801.1 (17855830..17857104	Malus domestica
MD15G1137600	14	32560231	NC_024252.1 (2371351..2373293)	Malus domestica
MD15G1061700	5	48068851	NC_041793.1 (18905905..18911038	Malus domestica
MD15G1333400	8	31666303	NC_041796.1 (3397877..3401201	Malus domestica
MD16G1200000	10	41841605	NC_041798.1 (4852509..4854441)	Malus domestica
Mapa_017194		772948	MUAA02000148.1	Marchantia paleacea
Mapa_016768		1581965	MUAA02000139.1	Marchantia paleacea
Mapa_014890		2225478	MUAA02000108.1	Marchantia paleacea
Mapa_014514		4600817	MUAA02000068.1	Marchantia paleacea
Mapa_014187		3119008	MUAA02000061.1	Marchantia paleacea
Mapa_014190		1575461	MUAA02000060.1	Marchantia paleacea
Mapa_014192		1841446	MUAA02000006.1	Marchantia paleacea
Mapa_014193		875316	MUAA02000103.1	Marchantia paleacea
Mapa_014195		1379221	MUAA02000098.1	Marchantia paleacea
Mapa_014196		1379221	MUAA02000098.1	Marchantia paleacea
Mapa_011878		1379221	MUAA02000098.1	Marchantia paleacea
Mapa_011901		1379221	MUAA02000098.1	Marchantia paleacea
Mapa_011453		1379221	MUAA02000098.1	Marchantia paleacea
Mapa_010820		1379221	MUAA02000098.1	Marchantia paleacea
Mapa_010273		2629209	MUAA02000074.1	Marchantia paleacea

Mapa_009996	2629209	MUAA02000074.1	Marchantia paleacea
Mapa_009823	2262681	MUAA02000070.1	Marchantia paleacea
Mapa_008095	2461926	MUAA02000064.1	Marchantia paleacea
Mapa_008163	5277378	MUAA02000049.1	Marchantia paleacea
Mapa_007988	5277378	MUAA02000049.1	Marchantia paleacea
Mapa_007989	430384	MUAA02000048.1	Marchantia paleacea
Mapa_007992	430384	MUAA02000048.1	Marchantia paleacea
Mapa_007995	430384	MUAA02000048.1	Marchantia paleacea
Mapa_007461	430384	MUAA02000048.1	Marchantia paleacea
Mapa_006341	2517113	MUAA02000041.1	Marchantia paleacea
Mapa_005629	2232704	MUAA02000032.1	Marchantia paleacea
Mapa_004408	2760609	MUAA02000024.1	Marchantia paleacea
Mapa_004804	7327666	MUAA02000018.1	Marchantia paleacea
Mapa_002052	7327666	MUAA02000018.1	Marchantia paleacea
Mapa_001420	8438675	MUAA02000010.1	Marchantia paleacea
Mapa_001056	430384	MUAA02000048.1	Marchantia paleacea
Mapa_014187	430384	MUAA02000048.1	Marchantia paleacea
Mapa_014190	430384	MUAA02000048.1	Marchantia paleacea
Mapa_014192	5277378	MUAA02000049.1	Marchantia paleacea
Mapa_014193	5277378	MUAA02000049.1	Marchantia paleacea
Mapa_014195	2461926	MUAA02000064.1	Marchantia paleacea
Mapa_014196	2262681	MUAA02000070.1	Marchantia paleacea
Mapa_011878	2629209	MUAA02000074.1	Marchantia paleacea
Mapa_011901	2629209	MUAA02000074.1	Marchantia paleacea
Mapa_011453	1379221	MUAA02000098.1	Marchantia paleacea
Mapa_010273	1379221	MUAA02000098.1	Marchantia paleacea
Mapa_008095	1379221	MUAA02000098.1	Marchantia paleacea
Mapa_008163	1379221	MUAA02000098.1	Marchantia paleacea
Mapa_007988	1379221	MUAA02000098.1	Marchantia paleacea
Mapa_007989	1379221	MUAA02000098.1	Marchantia paleacea
Mapa_007990	4427576	MUAA02000005.1	Marchantia paleacea
MARPO_0371s0001	43212	scaffold_278(19082..19465)	Marchantia polymorpha
MARPO_0278s0006	1167605	scaffold_65(117207..117497)	Marchantia polymorpha
MARPO_0199s0012	1444639	scaffold_47(1027671..1028015)	Marchantia polymorpha
MARPO_0199s0013	1444639	scaffold_47(1027671..1028015)	Marchantia polymorpha
MARPO_0199s0014	1444639	scaffold_47(1034923..1035273)	Marchantia polymorpha
MARPO_0199s0015	1444760	scaffold_46(959489..959863)	Marchantia polymorpha
MARPO_0199s0016	2356800	scaffold_13(2279870..2280115)	Marchantia polymorpha
MARPO_0199s0017	2356800	scaffold_13(2279870..2280115)	Marchantia polymorpha
MARPO_0199s0018	3206446	scaffold_4(1802068..1802448)	Marchantia polymorpha
MARPO_0199s0019	6192411	scaffold_1(534221..534790)	Marchantia polymorpha
MARPO_0199s0020	13382	scaffold_57(777462..776515)	Marchantia polymorpha
MARPO_0139s0012	13382	scaffold_57(870235..870882)	Marchantia polymorpha
MARPO_0083s0003	182516	scaffold_199(119069..119716)	Marchantia polymorpha

MARPO_0082s0040		182516	scaffold_199(122601..123248)	Marchantia polymorpha
MARPO_0079s0014		182516	scaffold_199(132408..133055)	Marchantia polymorpha
MARPO_0074s0027		182516	scaffold_199(122601..123248)	Marchantia polymorpha
MARPO_0068s0047		182516	scaffold_199(137520..136873)	Marchantia polymorpha
MARPO_0068s0071		182516	scaffold_199(137520..136873)	Marchantia polymorpha
MARPO_0068s0087		182516	scaffold_199(150933..151580)	Marchantia polymorpha
MARPO_0066s0050		1299771	scaffold_57(870235..870882)	Marchantia polymorpha
MARPO_0065s0010		460349	scaffold_139(241532..242179)	Marchantia polymorpha
MARPO_0057s0073		943319	scaffold_83(52016..52534)	Marchantia polymorpha
MARPO_0057s0082		948950	scaffold_82(394578..394264)	Marchantia polymorpha
MARPO_0057s0083		1121589	scaffold_68(393888..393469)	Marchantia polymorpha
MARPO_0047s0106		1060017	scaffold_74(372987..373406)	Marchantia polymorpha
MARPO_0047s0106		182516	scaffold_199(132399..133052)	Marchantia polymorpha
MARPO_0047s0108		182516	scaffold_199(132408133055)	Marchantia polymorpha
MARPO_0046s0095		1121589	scaffold_68(775738..774716)	Marchantia polymorpha
MARPO_0026s0025		182516	scaffold_199(10301..9609)	Marchantia polymorpha
MARPO_0013s0199		5297721	scaffold_66(434044..434658)	Marchantia polymorpha
MARPO_0013s0199		1299771	scaffold_57(870235..870882)	Marchantia polymorpha
MARPO_0011s0076		1299771	scaffold_57(870235..870882)	Marchantia polymorpha
MARPO_0008s0015		1824988	scaffold_26(359592..360245)	Marchantia polymorpha
MARPO_0006s0261		2578647	scaffold_11(769823..769281)	Marchantia polymorpha
MARPO_0004s0164		2905077	scaffold_8(161191..160190)	Marchantia polymorpha
MARPO_0001s0025		2946544	scaffold_6(2435081..2435512)	Marchantia polymorpha
MARPO_0001s0043		6192411	scaffold_1(326591..325575)	Marchantia polymorpha
MARPO_0139s0012		13382	scaffold_371(9204..10636)	Marchantia polymorpha
MARPO_0083s0003		182516	scaffold_199(115615..116902)	Marchantia polymorpha
MARPO_0082s0040		182516	scaffold_199(118628..119934)	Marchantia polymorpha
MARPO_0079s0014		182516	scaffold_199(122017..123714)	Marchantia polymorpha
MARPO_0074s0027		182516	scaffold_199(131853..133502)	Marchantia polymorpha
MARPO_0068s0047		182516	scaffold_199(134347..135905)	Marchantia polymorpha
MARPO_0068s0071		182516	scaffold_199(136763..138209)	Marchantia polymorpha
MARPO_0068s0087		182516	scaffold_199(149819..152095)	Marchantia polymorpha
MARPO_0066s0050		182516	scaffold_199(153529..154870)	Marchantia polymorpha
MARPO_0065s0010		182516	scaffold_199(156038..157448)	Marchantia polymorpha
MARPO_0057s0073		460349	scaffold_139(240692..242447)	Marchantia polymorpha
MARPO_0057s0082		943319	scaffold_83(51103..53043)	Marchantia polymorpha
MARPO_0057s0083		948950	scaffold_82(393667..395371)	Marchantia polymorpha
MARPO_0047s0106		974370	scaffold_79(129472..>129924)	Marchantia polymorpha
MARPO_0047s0106		1060017	scaffold_74(37197..38647)	Marchantia polymorpha
MARPO_0047s0108		1121589	scaffold_68(44069..45386)	Marchantia polymorpha
MARPO_0046s0095		1121589	scaffold_68(45968..47974)	Marchantia polymorpha
LOC_Os01g14410.1	1	43270923	NC_029256.1 (8061702..8062895)	Oryza sativa
LOC_Os01g64960.1	1	43270923	NC_029256.1 (37696783..37699583)	Oryza sativa
LOC_Os01g40710.1	1	43270923	NC_029256.1 (23003511..23004997)	Oryza sativa

LOC_Os02g03330.1	2	35937250	NC_029257.1 (1344644..1346980)	Oryza sativa
LOC_Os04g54630.1	4	35502694	NC_029259.1 (32480874..32482346)	Oryza sativa
LOC_Os04g59440.1	4	35502694	NC_029259.1 (35337051..35338194)	Oryza sativa
LOC_Os05g22730.1	5	29958434	NC_029260.1 (12895903..12896826)	Oryza sativa
LOC_Os05g29760.2	5	29958434	NC_029260.1 (17209577..17216385)	Oryza sativa
LOC_Os07g08150.1	7	29697621	NC_029262.1 (4143302..4144467)	Oryza sativa
LOC_Os07g08160.1	7	29697621	NC_029262.1 (4145833..4146869)	Oryza sativa
LOC_Os09g12560.1	9	23012720	NC_029264.1 (7198619..7204750)	Oryza sativa
LOC_Os11g13850.1	11	29021106	NC_029266.1 (7639475..7642985	Oryza sativa
LOC_Os11g12830.1	11	29021106	NC_029266.1 (7639475..7642985	Oryza sativa
LOC_Os12g29570.1	12	27531856	NC_029267.1 (17624380..17625450)	Oryza sativa
LOC_Os01g52240.1	1	43270923	NC_029256.1 (30030985..30032087)	Oryza sativa
LOC_Os01g41710.1	1	43270923	NC_029256.1 (23607346..23608505)	Oryza sativa
LOC_Os02g52650.1	2	35937250	NC_029257.1 (32199540..32201717	Oryza sativa
LOC_Os02g10390.1	2	35937250	NC_029257.1 (5468647..5470491	Oryza sativa
LOC_Os03g39610.1	3	36413819	NC_029258.1 (21999947..22001241)	Oryza sativa
LOC_Os06g21590.1	6	31248787	NC_029261.1 (12452660..12454083)	Oryza sativa
LOC_Os07g37550.1	7	29697621	NC_029262.1 (22487424..22488722)	Oryza sativa
LOC_Os07g37240.1	7	29697621	NC_029262.1 (22316313..22317929	Oryza sativa
LOC_Os07g38960.1	7	29697621	NC_029262.1 (23357688..23359391)	Oryza sativa
LOC_Os08g33820.1	8	28443022	NC_029263.1 (21171276..21172675	Oryza sativa
LOC_Os09g12540.1	9	23012720	NC_029264.1 (7185163..7188147)	Oryza sativa
LOC_Os09g17740.1	9	23012720	NC_029264.1 (10845683..10846965)	Oryza sativa
LOC_Os09g26810.1	9	23012720	NC_029264.1 (16287868..16290863)	Oryza sativa
OT_ostta02g03930	2	1074072	NC_014427.2 (769098..769463)	Ostreococcus tauri
OT_ostta03g01980	3	997348	NC_014428.2 (324476..325697)	Ostreococcus tauri
OT_ostta11g02670	11	595839	NC_014436.2 (480260..481873)	Ostreococcus tauri
OT_ostta02g03330	2	1074072	NC_014427.2 (649631..650596)	Ostreococcus tauri
OT_ostta02g04800	2	1074072	NC_014427.2 (945986..946918)	Ostreococcus tauri
OT_ostta14g00065	14	547765	NC_014439.2 (202558..203992)	Ostreococcus tauri
OT_ostta18g01230	18	345706	NC_014443.2 (223244..224356)	Ostreococcus tauri
OT_ostta05g03830	5	835544	NC_014430.2 (631907..632863)	Ostreococcus tauri
OT_ostta09g04420	9	713054	NC_014434.2 (694700..695618	Ostreococcus tauri
OT_ostta11g00990	11	595839	NC_014436.2 (190924..192012)	Ostreococcus tauri
OT_ostta14g00700	14	547765	NC_014439.2 (117191..121510)	Ostreococcus tauri
OT_ostta16g02310	16	528281	NC_014441.2 (443688..444918)	Ostreococcus tauri
WP_014605551.1		4586378	NC_017554.1(1882537..1883499)	Pantoea ananatis
XP_008797389.1	6	18596258	NC_052397.1（3659179..3664461）	Phoenix dactylifera
XP_008784592.1	6	18596258	NC_052397.1（6268813..6270731）	Phoenix dactylifera
XP_008786444.1	14	24628924	NC_052405.19308321..9322459）	Phoenix dactylifera
XP_017699741.1	14	24628924	NC_052405.1（9308321..9322459）	Phoenix dactylifera
XP_038986725.1	1	40814151	NC_052392.1 (26659188..26660869)	Phoenix dactylifera
XP_008783427.2	1	40814151	NC_052392.1 (35028801..35030874	Phoenix dactylifera
XP_008801597.2	3	24755689	NC_052394.1 (135891..138846	Phoenix dactylifera

XP_008806764.2	4	33281721	NC_052395.1 (18318949..18320195	Phoenix dactylifera
XP_038981698.1	4	33281721	NC_052395.1 (18713292..18720537)	Phoenix dactylifera
XP_008786198.2	5	18619412	NC_052396.1 (2879435..2882092)	Phoenix dactylifera
XP_008783815.1	6	18596258	NC_052397.1 (4990151..4991410)	Phoenix dactylifera
XP_038983214.1	6	18596258	NC_052397.1 (6158247..6159506	Phoenix dactylifera
XP_008782823.1	7	16639383	NC_052398.1 (4659539..4667369)	Phoenix dactylifera
XP_026658469.1	7	16639383	NC_052398.1 (4659539..4667369)	Phoenix dactylifera
XP_008779641.1	9	22757669	NC_052400.1 (16011501..16012615	Phoenix dactylifera
XP_008777380.2	10	15825318	NC_052401.1 (14936123..14947092)	Phoenix dactylifera
XP_008781977.1	10	15825318	NC_052401.1 (4186192..4190012	Phoenix dactylifera
XP_026663797.2	10	15825318	NC_052401.1 (8690585..8711734	Phoenix dactylifera
XP_008800689.2	11	29487722	NC_052402.1 (10835031..10836045	Phoenix dactylifera
XP_008800395.1	11	29487722	NC_052402.1 (16690713..16692039	Phoenix dactylifera
XP_008800353.2	11	29487722	NC_052402.1 (16877654..16881209)	Phoenix dactylifera
XP_008808729.3	11	29487722	NC_052402.1 (3267498..3268599)	Phoenix dactylifera
XP_008784474.2	11	29487722	NC_052402.1 (9631281..9633950	Phoenix dactylifera
XP_008805305.2	14	24628924	NC_052405.1 (3936385..3938587	Phoenix dactylifera
XP_038989517.1	14	24628924	NC_052405.1 (9603011..9607472)	Phoenix dactylifera
XP_008808974.1	14	24628924	NC_052405.1 (9911254..9915077	Phoenix dactylifera
XP_008797264.2	15	12030914	NC_052406.1 (4985092..4990785)	Phoenix dactylifera
XP_008790804.1	15	12030914	NC_052406.1 (6968709..6970642	Phoenix dactylifera
XP_008802578.1	15	12030914	NC_052406.1 (8000300..8001608	Phoenix dactylifera
XP_008787839.2	15	12030914	NC_052406.1 (9139982..9148048	Phoenix dactylifera
XP_008777102.3	16	13553361	NC_052407.1 (4654201..4656900	Phoenix dactylifera
XP_026657330.2	17	16126437	NC_052408.1 (3852818..3857962)	Phoenix dactylifera
XP_008807305.1	18	9812533	NC_052409.1 (56577..61270)	Phoenix dactylifera
XP_026665447.1	18	9812533	NC_052409.1 (56577..61270)	Phoenix dactylifera
XP_008810536.2		370528	NW_008246808.1 (81953..83657	Phoenix dactylifera
XP_008786056.2		4728343	NW_024067666.1(3918640..3920967)	Phoenix dactylifera
XP_026659405.2		4728343	NW_024067666.13418540..3420767)	Phoenix dactylifera
XP_008805513.1		532880	NW_024067797.1 (379847..381555	Phoenix dactylifera
XP_038975642.1		301455	NW_024068001.1 (94041..97852	Phoenix dactylifera
XP_038975643.1		301455	NW_024068001.1 (94041..97852	Phoenix dactylifera
XP_008809318.2		267500	NW_024068064.1 (177331..178909	Phoenix dactylifera
XP_008777764.1		241885	NW_024068138.1(190859..196630)	Phoenix dactylifera
Pp3c2_31600V3.1.p	1	30242098	NC_037253.1 (5318802..5323322)	Physcomitrium patens
Pp3c1_7090V3.1.p	1	30242098	NC_037253.1 (5318802..5323322)	Physcomitrium patens
Pp3c1_26740V3.1.p	1	30242098	NC_037253.1 (19404869..19406731)	Physcomitrium patens
Pp3c2_30980V3.1.p	2	25998084	NC_037254.1 (20979618..20984180	Physcomitrium patens
Pp3c2_31600V3.1.p	2	25998084	NC_037254.1 (18132439..18134832	Physcomitrium patens
Pp3c2_19700V3.1.p	2	25998084	NC_037254.1 (12631704..12633104	Physcomitrium patens
Pp3c11_6350V3.1.p	11	17494229	NC_037263.1 (3964187..3968768)	Physcomitrium patens
Pp3c14_9190V3.1.p	14	17248380	NC_037266.1 (5895112..5896940	Physcomitrium patens
Pp3c14_9180V3.1.p	14	17248380	NC_037266.1 (5890396..5892341	Physcomitrium patens

Pp3c20_23430V3.1.p	20	15486712	NC_037272.1 (15174432..15176223)	Physcomitrium patens
Pp3c22_8010V3.1.p	22	15354560	NC_037274.1 (4891796..4893430)	Physcomitrium patens
Pp3c1_7091V3.1.p	2	25998084	NC_037254.1 (20690681..20693172	Physcomitrium patens
Pp3c2_36380V3.1.p	2	25998084	NC_037254.1 (24510439..24513085	Physcomitrium patens
Pp3c2_36380V3.1.p	2	25998084	NC_037254.1 (24645745..24648391	Physcomitrium patens
Pp3c2_36380V3.1.p	2	25998084	NC_037254.1 (24834236..24835691	Physcomitrium patens
Pp3c2_36380V3.1.p	2	25998084	NC_037254.1 (24756129..24757624	Physcomitrium patens
Pp3c2_36380V3.1.p	2	25998084	NC_037254.1 (24534490..24536127)	Physcomitrium patens
Pp3c2_35930V3.1.p	2	25998084	NC_037254.1 (24320584..24322050)	Physcomitrium patens
Pp3c3_4100V3.1.p	3	25551832	NC_037255.1 (2507435..2508854)	Physcomitrium patens
Pp3c3_12510V3.1.p	3	25551832	NC_037255.1 (8668396..8670314)	Physcomitrium patens
Pp3c3_4140V3.1.p	3	25551832	NC_037255.1 (2540398..2541753	Physcomitrium patens
Pp3c4_11854V3.1.p	4	22344759	NC_037256.1 (8338864..8341993)	Physcomitrium patens
Pp3c4_17240V3.1.p	4	22344759	NC_037256.1 (11896944..11898455	Physcomitrium patens
Pp3c4_29270V3.1.p	4	22344759	NC_037256.1 (20172225..20175048	Physcomitrium patens
Pp3c4_29150V3.4.p	4	22344759	NC_037256.1 (20172225..20175048	Physcomitrium patens
Pp3c4_5690V3.1.p	4	22344759	NC_037256.1 (3954892..3956451)	Physcomitrium patens
Pp3c4_5690V3.1.p	4	22344759	NC_037256.1 (3957440..3958941	Physcomitrium patens
Pp3c4_28410V3.1.p	4	22344759	NC_037256.1 (19526322..19531862)	Physcomitrium patens
Pp3c4_28370V3.1.p	4	22344759	NC_037256.1 (19523444..19525576)	Physcomitrium patens
Pp3c4_29270V3.1.p	4	22344759	NC_037256.1 (20269866..20272689)	Physcomitrium patens
Pp3c4_29270V3.1.p	4	22344759	NC_037256.1 (20269866..20272689)	Physcomitrium patens
Pp3c5_22920V3.1.p	5	20681290	NC_037257.1 (16084250..16086087)	Physcomitrium patens
Pp3c5_7150V3.1.p	5	20681290	NC_037257.1 (5022791..5024497	Physcomitrium patens
Pp3c5_7150V3.1.p	5	20681290	NC_037257.1 (5022791..5024498	Physcomitrium patens
Pp3c5_7150V3.1.p	5	20681290	NC_037257.1 (5022791..5024499	Physcomitrium patens
Pp3c6_12510V3.1.p	6	19533230	NC_037258.1 (8056848..8058174)	Physcomitrium patens
Pp3c6_22090V3.1.p	6	19533230	NC_037258.1 (14183704..14186176	Physcomitrium patens
Pp3c6_6830V3.1.p	6	19533230	NC_037258.1 (4084477..4086399)	Physcomitrium patens
Pp3c10_25415V3.1.p	10	17530623	NC_037262.1 (17081446..17083591	Physcomitrium patens
Pp3c10_25360V3.1.p	6	19533230	NC_037258.1 (4084477..4086399)	Physcomitrium patens
Pp3c10_25450V3.1.p	10	17530623	NC_037262.1 (17205065..17206940)	Physcomitrium patens
Pp3c10_3020V3.1.p	10	17530623	NC_037262.1 (2387424..2388837	Physcomitrium patens
Pp3c12_25430V3.1.p	12	17435539	NC_037264.1 (16711249..16713063)	Physcomitrium patens
Pp3c12_25430V3.1.p	12	17435539	NC_037264.1 (16735957..16737784	Physcomitrium patens
Pp3c12_19200V3.1.p	12	17435539	NC_037264.1 (12375763..12378382	Physcomitrium patens
Pp3c12_19200V3.1.p	12	17435539	NC_037264.1 (12375763..12378382	Physcomitrium patens
Pp3c12_19200V3.1.p	12	17435539	NC_037264.1 (12375763..12378382	Physcomitrium patens
Pp3c12_19200V3.1.p	12	17435539	NC_037264.1 (12375763..12378382	Physcomitrium patens
Pp3c12_19200V3.1.p	12	17435539	NC_037264.1 (12375763..12378382	Physcomitrium patens
Pp3c13_5930V3.1.p	13	17250819	NC_037265.1 (3934666..3935883	Physcomitrium patens
Pp3c13_7900V3.1.p	13	17250819	NC_037265.1 (5239725..5241323)	Physcomitrium patens
Pp3c13_14980V3.1.p	13	17250819	NC_037265.1 (10931790..10933687	Physcomitrium patens
Pp3c16_16040V3.1.p	16	16572167	NC_037268.1 (9910325..9912773)	Physcomitrium patens

Pp3c18_8100V3.1.p	18	15735161	NC_037270.1 (5698799..5700675	Physcomitrium patens
Potri.001G210000.1.p	1	49788581	NC_037285.1 (21067273..21068553	Populus trichocarpa
Potri.001G301100.2.p	1	49788581	NC_037285.1 (30500511..30502514)	Populus trichocarpa
Potri.002G065000.1.p	2	25242375	NC_037286.1 (4376415..4378586)	Populus trichocarpa
Potri.002G083500.4.p	2	25242375	NC_037286.1 (5849372..5851329)	Populus trichocarpa
Potri.002G221400.1.p	2	25242375	NC_037286.1 (20839286..20841155)	Populus trichocarpa
Potri.003G171500.1.p	2	25242375	NC_037286.1 (14958412..14959358)	Populus trichocarpa
Potri.T125006.1.p	3	21678634	NC_037287.1 (18154970..18156665)	Populus trichocarpa
Potri.005G239300.1.p	5	24981103	NC_037289.1 (24586875..24587931)	Populus trichocarpa
Potri.005G258600.1.p	5	24981103	NC_037289.1 (6249088..6250880)	Populus trichocarpa
Potri.005G239200.1.p	5	24981103	NC_037289.1 (24586875..24587931)	Populus trichocarpa
Potri.006G099500.1.p	5	24981103	NC_037289.1 (25755683..25758544)	Populus trichocarpa
Potri.008G041000.1.p	5	24981103	NC_037289.1 (21269073..21271110	Populus trichocarpa
Potri.008G067300.1.p	5	24981103	NC_037289.1 (24584865..24585906	Populus trichocarpa
Potri.010G221100.4.p	6	27516652	NC_037290.1 (6737060..6737919	Populus trichocarpa
Potri.011G079500.1.p	6	27516652	NC_037290.1 (7645805..7646939	Populus trichocarpa
Potri.014G165100.1.p	6	27516652	NC_037290.1 (24214350..24218013	Populus trichocarpa
Potri.014G172400.1.p	8	19195260	NC_037292.1 (2336952..2338945	Populus trichocarpa
Potri.014G056800.1.p	8	19195260	NC_037292.1 (4082675..4084944	Populus trichocarpa
Potri.016G115200.1.p	8	19195260	NC_037292.1 (10695456..10696500	Populus trichocarpa
Potri.010G054400.1.p	8	19195260	NC_037292.1 (10703882..10705004	Populus trichocarpa
Potri.001G407100.1.p	8	19195260	NC_037292.1 (10708295..10709451	Populus trichocarpa
Potri.002G189300.1.p	10	22799081	NC_037294.1 (20608772..20610210)	Populus trichocarpa
Potri.003G083500.2.p	11	19288771	NC_037295.1 (7893753..7894770	Populus trichocarpa
Potri.006G139600.1.p	14	17801709	NC_037298.1 (13078768..13097505	Populus trichocarpa
Potri.006G232100.2.p	14	17801709	NC_037298.1 (13984662..13986006	Populus trichocarpa
Potri.008G158033.2.p	16	14619816	NC_037300.1 (11963045..11964207	Populus trichocarpa
Potri.008G158166.2.p	19	15623655	NC_037303.1 (10796069..10798883	Populus trichocarpa
Potri.003G083500.2.p	5	24981103	NC_037289.1 (15755283..15755544)	Populus trichocarpa
Potri.019G073900.1.p	19	15623655	NC_037303.1 (8550078..8551988	Populus trichocarpa
Potri.T010900.5.p	6	27516652	NC_037290.1 (4645505..4647939	Populus trichocarpa
Potri.001G151300.2.p	5	24981103	NC_037289.1 (14784865..14785906	Populus trichocarpa
Potri.004G138400.1.p	1	49788581	NC_037285.1 (12481520..12483983	Populus trichocarpa
Potri.005G084200.1.p	1	49788581	NC_037285.1 (42958618..42959949	Populus trichocarpa
Potri.005G196100.1.p	19	15623655	NW_020167874.1 (50671..56965)	Populus trichocarpa
Potri.006G088200.1.p	15	15231745	NC_037299.1 (12714563..12716032)	Populus trichocarpa
Potri.008G157750.1.p	8	19195260	NC_037292.1 (10713976..10715122	Populus trichocarpa
Potri.008G157900.7.p	6	27516652	NC_037290.1 (11703115..11704744)	Populus trichocarpa
Potri.009G096900.1.p	3	21678634	NC_037287.1 (11100965..11105020)	Populus trichocarpa
Potri.005G258600.1.p	4	24140038	NC_037288.1 (16027280..16034160	Populus trichocarpa
Potri.008G041000.1.p	9	12987399	NC_037293.1 (8707082..8711770)	Populus trichocarpa
XP_002966860.1		3666672	NW_003314269.1(522482..524865)	Selaginella moellendorffii
XP_002973413.1		2100764	NW_003314282.1(1418609..1419821)	Selaginella moellendorffii
XP_002976494.1		1846953	NW_003314292.1(311153..312303)	Selaginella moellendorffii

XP_002976832.1		1840579	NW_003314293.1(560462..561583)	Selaginella moellendorffii
XP_002978841.1		1751465	NW_003314299.1(1461894..1463286)	Selaginella moellendorffii
XP_002980561.1		1506775	NW_003314306.1(568155..569305)	Selaginella moellendorffii
XP_002961149.2		6951972	NW_003314261.1(4850651..4853046)	Selaginella moellendorffii
XP_002963893.2		4340499	NW_003314264.1(4089864..4092033)	Selaginella moellendorffii
XP_002977290.2		4340499	NW_003314294.1（1416755..1417830）	Selaginella moellendorffii
XP_002988297.2		828699	NW_003314348.1(260956..263722)	Selaginella moellendorffii
XP_002992177.2		396342	NW_003314390.1(357335..358413)	Selaginella moellendorffii
XP_002993521.2		230390	NW_003314422.1(5832..6657)	Selaginella moellendorffii
XP_002960494.1		6951972	NW_003314261.1(4789661..4790848)	Selaginella moellendorffii
XP_002964438.1		4143896	NW_003314265.1(1316044..1317324)	Selaginella moellendorffii
XP_002966149.1		3908038	NW_003314268.1(517796..518967)	Selaginella moellendorffii
XP_002974259.1		1997012	NW_003314285.1(936767..938034)	Selaginella moellendorffii
XP_002975356.1		1968041	NW_003314288.1(1844243..1845536)	Selaginella moellendorffii
XP_002976428.1		1905289	NW_003314291.1(970377..972042)	Selaginella moellendorffii
XP_002980072.1		1658729	NW_003314304.1(364061..365404)	Selaginella moellendorffii
XP_002982144.1		1451693	NW_003314313.1(78150..79285)	Selaginella moellendorffii
XP_002984009.1		1246421	NW_003314321.1(413034..414283)	Selaginella moellendorffii
XP_002984998.1		1132267	NW_003314326.1(647229..648750)	Selaginella moellendorffii
XP_002985629.1		1032227	NW_003314330.1(500697..501898)	Selaginella moellendorffii
XP_002985890.1		997799	NW_003314331.1(60068..61388)	Selaginella moellendorffii
XP_002986188.1		981669	NW_003314333.1(853176..854581)	Selaginella moellendorffii
Solyc12g006140.2.1	2	55977580	NC_015439.2 (40525924..40526861)	Solanum lycopersicum
Solyc03g094000.3.1	4	66557038	NC_015441.2 (66402402..66411410)	Solanum lycopersicum
Solyc04g053130.4.1	12	68126176	NC_015449.3 (693012..694548	Solanum lycopersicum
Solyc04g071930.4.1	10	65633393	NC_015447.3 (913432..917016)	Solanum lycopersicum
Solyc04g082920.4.1	6	49794276	NC_015443.3 (38438906..38440844)	Solanum lycopersicum
Solyc05g056050.3.1	10	65633393	NC_015447.2 (920029..923613)	Solanum lycopersicum
Solyc07g022900.4.1	1	98455869	NC_015438.3 (93221230..93222360)	Solanum lycopersicum
Solyc07g063600.3.1	1	98455869	NC_015438.2 (93326951..93328081)	Solanum lycopersicum
Solyc08g007180.3.1	9	72906345	NC_015446.3 (68834940..68839494	Solanum lycopersicum
Solyc08g065480.3.1	2	55977580	NC_015439.3 (41093694..41094631)	Solanum lycopersicum
Solyc08g077880.3.1	3	72290146	NC_015440.2 (65491576..65492818)	Solanum lycopersicum
Solyc09g014520.3.1	3	72290146	NC_015440.3 (66975786..66977028)	Solanum lycopersicum
Solyc09g011010.3.1	3	72290146	NC_015440.3 (540964..541913)	Solanum lycopersicum
Solyc10g084140.2.1	3	72290146	NC_015440.2 (540960..541909)	Solanum lycopersicum
Solyc11g042640.2.1	6	49794276	NC_015443.3 (43549398..43550509)	Solanum lycopersicum
Solyc12g009200.2.1	1	98455869	NC_015438.3 (95026332..95028476	Solanum lycopersicum
Solyc12g011280.2.1	3	72290146	NC_015440.3 (57142219..57143964	Solanum lycopersicum
Solyc12g056620.2.1	4	66557038	NC_015441.3 (51662128..51664403	Solanum lycopersicum
Solyc03g005760.1.1	4	66557038	NC_015441.3 (59030916..59033118	Solanum lycopersicum
Solyc05g018650.4.1	4	66557038	NC_015441.3 (66488506..66497506)	Solanum lycopersicum
Solyc08g065480.3.1	7	68175699	NC_015444.2 (23224792..23226435)	Solanum lycopersicum
Solyc10g084140.2.1	7	68175699	NC_015444.3 (23219661..23221300)	Solanum lycopersicum

Solyc12g006140.2.1	9	72906345	NC_015446.2 (6145925..6147802	Solanum lycopersicum
Solyc06g060340.3.1	8	65987440	NC_015445.3 (1742194..1745604	Solanum lycopersicum
Solyc12g011450.2.1	8	65987440	NC_015445.3 (53650255..53657826	Solanum lycopersicum
Solyc10g006230.3.1	8	65987440	NC_015445.3 (61909498..61911417	Solanum lycopersicum
Solyc01g105050.3.1	9	72906345	NC_015446.3 (6146164..6148033	Solanum lycopersicum
Solyc01g105030.3.1	9	72906345	NC_015446.3 (4358433..4359627)	Solanum lycopersicum
Solyc05g056050.3.1	10	65633393	NC_015447.3 (63907852..63919553	Solanum lycopersicum
Solyc09g082690.3.1	11	56597135	NC_015448.3 (35824080..35827547	Solanum lycopersicum
Solyc02g071030.2.1	12	68126176	NC_015449.2 (4139322..4140948	Solanum lycopersicum
Solyc02g070980.1.1	12	68126176	NC_015449.3 (4139334..4141073	Solanum lycopersicum
Solyc03g115900.4.1	12	68126176	NC_015449.3 (63542038..63544188	Solanum lycopersicum
Solyc03g005780.3.1	10	65633393	NC_015447.3 (63907852..63919553	Solanum lycopersicum
Solyc03g005760.1.1	12	68126176	NC_015449.2 (693097..694633	Solanum lycopersicum
Solyc06g069730.3.1	5	66723567	NC_015442.3 (22690079..22709157	Solanum lycopersicum
Solyc01g107660.3.1	8	65987440	NC_015445.3 (53650255..53657826	Solanum lycopersicum
Solyc03g094000.3.1	9	72906345	NC_015446.3 (68834940..68839494	Solanum lycopersicum
Solyc09g082690.3.1	3	72290146	NC_015440.3 (57142219..57143964	Solanum lycopersicum
Solyc09g082700.2.1	3	72290146	NC_015440.3 (57142219..57143964	Solanum lycopersicum
Sobic.001G177000.1.p	9	59416394	NC_012878.2 (5639847..5640791)	Sorghum bicolor
Sobic.002G159100.1.p	9	59416394	NC_012878.2 (57389148..57390211)	Sorghum bicolor
Sobic.002G288300.1.p	6	61277060	NC_012875.2 (57694711..57696098)	Sorghum bicolor
Sobic.002G289100.1.p	6	61277060	NC_012875.2 (59864461..59865851)	Sorghum bicolor
Sobic.002G339200.2.p	5	71854669	NC_012874.2 (12277886..12279910	Sorghum bicolor
Sobic.002G033800.1.p	4	68658214	NC_012873.2 (2006032..2008116)	Sorghum bicolor
Sobic.002G052000.2.p	4	68658214	NC_012873.2 (4564190..4565419)	Sorghum bicolor
Sobic.002G338000.1.p	4	68658214	NC_012873.2 (526716..532557	Sorghum bicolor
Sobic.003G203000.1.p	4	68658214	NC_012873.2 (53230827..53236490	Sorghum bicolor
Sobic.003G209900.1.p	4	68658214	NC_012873.2 (64612233..64614586	Sorghum bicolor
Sobic.003G209900.1.p	3	74386277	NC_012872.2 (53289694..53291246)	Sorghum bicolor
Sobic.003G370000.1.p	3	74386277	NC_012872.2 (54181152..54182343)	Sorghum bicolor
Sobic.004G024600.1.p	3	74386277	NC_012872.2 (54181152..54182343)	Sorghum bicolor
Sobic.004G056900.1.p	3	74386277	NC_012872.2 (68596201..68598627)	Sorghum bicolor
Sobic.004G006200.1.p	2	77742459	NC_012871.2 (49231578..49252730)	Sorghum bicolor
Sobic.004G308700.1.p	2	77742459	NC_012871.2 (49269271..49279919)	Sorghum bicolor
Sobic.005G087000.1.p	2	77742459	NC_012871.2 (66709337..66710780)	Sorghum bicolor
Sobic.006G235300.1.p	2	77742459	NC_012871.2 (66797908..66798684)	Sorghum bicolor
Sobic.006G264201.1.p	2	77742459	NC_012871.2 (66709337..66710780)	Sorghum bicolor
Sobic.009G055800.2.p	2	77742459	NC_012871.2 (3103068..3104289	Sorghum bicolor
Sobic.009G234600.1.p	2	77742459	NC_012871.2 (4934989..4935985	Sorghum bicolor
Sobic.002G033800.1.p	2	77742459	NC_012871.2 (66797908..66798684)	Sorghum bicolor
Sobic.002G052100.1.p	1	80884392	NC_012870.2 (14892326..14893532	Sorghum bicolor
Sobic.002G352100.1.p	10	61233695	NC_012879.2 (52995719..52997456)	Sorghum bicolor
Sobic.002G352100.1.p	10	61233695	NC_012879.2（52995719..52997456）	Sorghum bicolor
Sobic.002G215000.2.p	2	77742459	NC_012871.2 (3103068..3104289	Sorghum bicolor

Sobic.002G159300.2.p	2	77742459	NC_012871.2 (4938721..4939660	Sorghum bicolor
Sobic.003G409700.2.p	2	77742459	NC_012871.2 (71556400..71557929)	Sorghum bicolor
Sobic.004G179400.2.p	2	77742459	NC_012871.2 (60709223..60710923)	Sorghum bicolor
Sobic.004G006200.2.p	3	74386277	NC_012872.2 (71713067..71715832)	Sorghum bicolor
Sobic.007G136900.1.p	4	68658214	NC_012873.2 (526716..532557	Sorghum bicolor
Sobic.010G189300.2.p	7	65505356	NC_012876.2 (56440053..56441675)	Sorghum bicolor
Sobic.010G189300.2.p	7	65505356	56440053..56441675	Sorghum bicolor
	LG0			
CY35_19G087000	1	32361881	CM038907.1	Sphagnum magellanicum
	LG0			
CY35_19G091800	1	32361881	CM038907.1	Sphagnum magellanicum
	LG0			
CY35_19G093000	1	32361881	CM038907.1	Sphagnum magellanicum
	LG0			
CY35_17G032000	1	32361881	CM038907.1	Sphagnum magellanicum
	LG0			
CY35_17G032200	2	36273875	CM038908.1	Sphagnum magellanicum
	LG0			
CY35_17G077400	2	36273875	CM038908.1	Sphagnum magellanicum
	LG0			
CY35_17G077600	2	36273875	CM038908.1	Sphagnum magellanicum
	LG0			
CY35_16G009700	2	36273875	CM038908.1	Sphagnum magellanicum
	LG0			
CY35_15G003500	2	36273875	CM038908.1	Sphagnum magellanicum
	LG0			
CY35_15G035200	2	36273875	CM038908.1	Sphagnum magellanicum
	LG0			
CY35_14G016200	2	36273875	CM038908.1	Sphagnum magellanicum
	LG0			
CY35_14G068100	3	25211390	CM038909.1	Sphagnum magellanicum
	LG0			
CY35_14G071700	3	25211390	CM038909.1	Sphagnum magellanicum
	LG0			
CY35_13G102000	3	25211390	CM038909.1	Sphagnum magellanicum
	LG0			
CY35_12G034600	3	25211390	CM038909.1	Sphagnum magellanicum
	LG0			
CY35_12G042800	4	26738934	CM038910.1	Sphagnum magellanicum
	LG0			
CY35_12G060700	4	26738934	CM038910.1	Sphagnum magellanicum
	LG0			
CY35_12G076200	4	26738934	CM038910.1	Sphagnum magellanicum
CY35_12G076200	LG0	25041821	CM038911.1	Sphagnum magellanicum

	5			
CY35_12G076200	LG05	25041821	CM038911.1	Sphagnum magellanicum
CY35_10G077300	LG05	25041821	CM038911.1	Sphagnum magellanicum
CY35_09G030400	LG05	25041821	CM038911.1	Sphagnum magellanicum
CY35_09G092300	LG05	25041821	CM038911.1	Sphagnum magellanicum
CY35_08G112600	LG06	26086143	CM038912.1	Sphagnum magellanicum
CY35_07G012600	LG06	26086143	CM038912.1	Sphagnum magellanicum
CY35_07G092300	LG06	26086143	CM038912.1	Sphagnum magellanicum
CY35_07G102300	LG07	20939185	CM038913.1	Sphagnum magellanicum
CY35_06G061300	LG07	20939185	CM038913.1	Sphagnum magellanicum
CY35_06G083100	LG07	20939185	CM038913.1	Sphagnum magellanicum
CY35_06G122500	LG08	25344393	CM038914.1	Sphagnum magellanicum
CY35_05G051700	LG09	20307503	CM038915.1	Sphagnum magellanicum
CY35_05G128200	LG09	20307503	CM038915.1	Sphagnum magellanicum
CY35_05G139900	LG10	18293271	CM038916.1	Sphagnum magellanicum
CY35_05G139900	LG12	20993935	CM038918.1	Sphagnum magellanicum
CY35_05G139900	LG12	20993935	CM038918.1	Sphagnum magellanicum
CY35_04G026600	LG12	20993935	CM038918.1	Sphagnum magellanicum
CY35_04G040600	LG12	20993935	CM038918.1	Sphagnum magellanicum
CY35_04G040600	LG12	20993935	CM038918.1	Sphagnum magellanicum
CY35_03G064800	LG12	20993935	CM038918.1	Sphagnum magellanicum
CY35_03G064800	LG13	23152942	CM038919.1	Sphagnum magellanicum
CY35_02G028100	LG1	20468628	CM038920.1	Sphagnum magellanicum

	4			
CY35_02G102200	LG1<br>4	20468628	CM038920.1	Sphagnum magellanicum
CY35_02G196700	LG1<br>4	20468628	CM038920.1	Sphagnum magellanicum
CY35_02G196800	LG1<br>5	18169471	CM038921.1	Sphagnum magellanicum
CY35_01G134000	LG1<br>5	18169471	CM038921.1	Sphagnum magellanicum
CY35_01G134100	LG1<br>6	18287404	CM038922.1	Sphagnum magellanicum
CY35_07G102300	LG1<br>7	19854503	CM038923.1	Sphagnum magellanicum
CY35_06G061300	LG1<br>7	19854503	CM038923.1	Sphagnum magellanicum
CY35_06G083100	LG1<br>7	19854503	CM038923.1	Sphagnum magellanicum
CY35_06G122500	LG1<br>7	19854503	CM038923.1	Sphagnum magellanicum
CY35_05G051700	LG1<br>9	17460353	CM038925.1	Sphagnum magellanicum
CY35_05G128200	LG1<br>9	17460353	CM038925.1	Sphagnum magellanicum
CY35_09G030400	LG1<br>9	17460353	CM038925.1	Sphagnum magellanicum
Thecc.06G154900.1.p	10	21822658	NC_030859.1 (989077..991442)	Theobroma cacao
Thecc.05G017200.1.p	9	38630433	NC_030858.1 (2611716..2614966)	Theobroma cacao
Thecc.02G208300.1.p	8	19593398	NC_030857.1 (382135..383744)	Theobroma cacao
Thecc.01G191400.1.p	8	19593398	NC_030857.1 (6480993..6482947	Theobroma cacao
Thecc.01G112400.1.p	8	19593398	NC_030857.1 (8837468..8839148	Theobroma cacao
Thecc.01G203200.1.p	8	19593398	NC_030857.1 (14824023..14828653	Theobroma cacao
Thecc.01G237100.1.p	7	21614486	NC_030856.1 (4139653..4140933)	Theobroma cacao
Thecc.02G106900.1.p	6	26303135	NC_030855.1 (22203833..22204909	Theobroma cacao
Thecc.02G141900.1.p	6	26303135	NC_030855.1 (22206371..22207357)	Theobroma cacao
Thecc.03G090600.1.p	5	39371879	NC_030854.1 (807131..808650)	Theobroma cacao
Thecc.03G240800.1.p	4	31929104	NC_030853.1 (28256132..28257134	Theobroma cacao
Thecc.04G238900.1.p	3	36364294	NC_030852.1 (21931081..21932391)	Theobroma cacao
Thecc.09G073800.2.p	3	36364294	NC_030852.1 (32087578..32090014	Theobroma cacao
Thecc.09G051900.1.p	2	41236440	NC_030851.1 (5399867..5401444	Theobroma cacao
Thecc.05G158800.1.p	2	41236440	NC_030851.1 (7388510..7389687	Theobroma cacao
Thecc.05G035600.1.p	2	41236440	NC_030851.1 (17060434..17062366	Theobroma cacao
Thecc.06G154800.1.p	1	37323695	NC_030850.1 (6004305..6006066)	Theobroma cacao
Thecc.07G070900.1.p	1	37323695	NC_030850.1 (13015546..13017114	Theobroma cacao
Thecc.08G158600.1.p	1	37323695	NC_030850.1 (14152053..14153593	Theobroma cacao

Thecc.08G007900.1.p	1	37323695	NC_030850.1 (21204419..21219868	Theobroma cacao
Thecc.08G125500.1.p	5	39371879	NC_030854.1 (27259300..27262085)	Theobroma cacao
Thecc.08G167600.1.p	5	39371879	NC_030854.1 (1617585..1618454)	Theobroma cacao
Thecc.08G174200.1.p	8	19593398	NC_030857.1 (16495191..16497814	Theobroma cacao
Thecc.08G174200.1.p	8	19593398	NC_030857.1 (14824023..14828653	Theobroma cacao
Thecc.10G020400.1.p	9	38630433	NC_030858.1 (3845879..3848128)	Theobroma cacao
Traes_5DL_F8A60AE39.7	1A	598660471	NC_057794.1 (507985679..507987919	Triticum aestivum
Traes_1AL_0F39673AF1.3	1A	598660471	NC_057794.1 (211441665..211450977	Triticum aestivum
Traes_1AL_06FED2723.1	2B	812755788	NC_057798.1 (757355997..757357257	Triticum aestivum
Traes_1AL_06FED2723.1	1B	700547350	NC_057795.1 (559833855..559834754	Triticum aestivum
Traes_1AL_06FED2723.1	1A	598660471	NC_057794.1 (500099399..500100465	Triticum aestivum
Traes_1AL_BEFAA871B.<br>1	1A	598660471	NC_057794.1 (507997911..507998835	Triticum aestivum
Traes_1AS_22266C808.4	3A	754128162	NC_057800.1 (392421440..392422726)	Triticum aestivum
Traes_1AL_6E5BA9C6F.1	1B	700547350	NC_057795.1 (559833855..559834754	Triticum aestivum
Traes_1AL_06FED2723.1	1A	598660471	NC_057794.1 (548921510..548922651	Triticum aestivum
Traes_1AL_0F39673AF1.3	1A	598660471	NC_057794.1 (548942987..548944126	Triticum aestivum
Traes_1AL_0F39673AF1.3	1A	598660471	NC_057794.1 (548988883..548989960	Triticum aestivum
Traes_1AL_0F39673AF1.3	1A	598660471	NC_057794.1 (549011784..549012925	Triticum aestivum
Traes_1AL_0F39673AF1.3	3B	851934019	NC_057801.1 (393460001..393461499)	Triticum aestivum
Traes_1AS_22266C808.4	1B	700547350	NC_057795.1 (559833855..559834754	Triticum aestivum
Traes_1BL_5AEE95D4C.1	3D	619618552	NC_057802.1 (291403313..291404872)	Triticum aestivum
Traes_1BL_5059FD5F8.1	1B	700547350	NC_057795.1 (559833855..559834754	Triticum aestivum
Traes_1AL_06FED2723.1	4A	754227511	NC_057803.1 (60333589..60334684)	Triticum aestivum
Traes_1AL_06FED2723.1	4A	754227511	NC_057803.1 (65467017..65468455)	Triticum aestivum
Traes_1AS_22266C808.4	4A	754227511	NC_057803.1 (534497116..534500972)	Triticum aestivum
Traes_2BL_CCD296233.1	4B	673810255	NC_057804.1 (93471591..93475377	Triticum aestivum
Traes_2BL_CCD296233.1	4B	673810255	NC_057804.1 (93471591..93475377	Triticum aestivum
Traes_2BL_CCD296233.1	4B	673810255	NC_057804.1 (470225353..470226334)	Triticum aestivum
Traes_3B_3B8AE232D.1	4B	673810255	NC_057804.1 (498231150..498232070)	Triticum aestivum
Traes_3B_D88E04B94.1	4D	518332611	NC_057805.1 (62598189..62602364)	Triticum aestivum
Traes_3B_3B8AE232D.1	4D	518332611	NC_057805.1 (383755680..383756601)	Triticum aestivum
Traes_3B_D88E04B94.1	4D	518332611	NC_057805.1 (402883897..402884807)	Triticum aestivum
Traes_3B_3B8AE232D.1	5A	713360525	NC_057806.1 (436716000..436727137)	Triticum aestivum
Traes_3B_D88E04B94.1	5A	713360525	NC_057806.1 (375601103..375606784)	Triticum aestivum
Traes_4AS_D13EBCE43.2	5A	713360525	NC_057806.1 (436476783..436477747)	Triticum aestivum
Traes_4AS_9C1CB8D5A.<br>9	5A	713360525	NC_057806.1 (436684878..436685625)	Triticum aestivum
Traes_4BS_1978E4612.1	5B	714697677	NC_057807.1 (324491258..324497100)	Triticum aestivum
Traes_4BS_1978E4612.1	5B	714697677	NC_057807.1 (394991132..394992030)	Triticum aestivum
Traes_4BS_1978E4612.1	5B	714697677	NC_057807.1 (395097570..395098079)	Triticum aestivum
Traes_4BL_C73920853.2	5B	714697677	NC_057807.1 (394770900..394771625	Triticum aestivum
Traes_4AS_D13EBCE43.2	5B	714697677	NC_057807.1 (395138126..395138503)	Triticum aestivum
Traes_4DS_0A0A9BE5B.1	1B	700547350	NC_057795.1 (236101493..236114494)	Triticum aestivum

Traes_4AS_9C1CB8D5A.9	5D	569951140	NC_057808.1 (285766489..285771906)	Triticum aestivum
Traes_4AS_D13EBCE43.2	5D	569951140	NC_057808.1 (337976390..337977146	Triticum aestivum
Traes_5DL_60524C948.2	6A	622669697	NC_057809.1 (616245184..616246325)	Triticum aestivum
Traes_5DL_5F223C713.2	6A	622669697	NC_057809.1 (55280998..55283060)	Triticum aestivum
Traes_5DL_60524C948.2	6B	731188232	NC_057810.1 (100896608..100898411	Triticum aestivum
Traes_5DL_3EE9B0FCD.2	1B	700547350	NC_057795.1 (559723716..559724683	Triticum aestivum
Traes_5BL_FF9AA4B58.1	1B	700547350	NC_057795.1 (559833855..559834754	Triticum aestivum
Traes_5DL_F8A60AE39.7	6B	731188232	NC_057810.1 (721520784..721528200)	Triticum aestivum
Traes_5DL_F8A60AE39.3	6D	495380293	NC_057811.1 (488407937..488409118)	Triticum aestivum
Traes_5BL_88FA5246A.2	6D	495380293	NC_057811.1 (56126733..56128878)	Triticum aestivum
Traes_5DL_60524C948.2	7A	744491536	NC_057812.1 (183077508..183078387	Triticum aestivum
Traes_5DL_5F223C713.2	7B	764072961	NC_057813.1 (143904563..143905486	Triticum aestivum
Traes_5DL_3EE9B0FCD.2	1A	598660471	NC_057794.1 (507794086..507800294	Triticum aestivum
Traes_6AL_01B48F86C.1	1A	598660471	NC_057794.1 (507813842..507814722	Triticum aestivum
Traes_6AS_D921D884D.1	1A	598660471	NC_057794.1 (507978877..507979777	Triticum aestivum
Traes_6AS_D921D884D.1	7D	642921167	NC_057814.1 (177613247..177614117	Triticum aestivum
Traes_6AL_01B48F86C.1	1D	498638509	NC_057796.1 (412742534..412743452	Triticum aestivum
Traes_6DL_EAF694645.1	1B	700547350	NC_057795.1 (559833855..559834754	Triticum aestivum
Traes_6AS_D921D884D.1	1A	598660471	NC_057794.1 (547012784..547012925	Triticum aestivum
Traes_5DL_60524C948.2		2418	NW_025247408.1 (2052..2368)	Triticum aestivum
Traes_5DL_60524C948.2	1D	498638509	NC_057796.1 (152549830..152560490)	Triticum aestivum
Traes_5DL_60524C948.2	1B	700547350	NC_057795.1 (559833855..559834754	Triticum aestivum
VIT_203s0180g00110.1	5	25021643	NC_012011.3 (5771417..5772199	Vitis vinifera
VIT_204s0008g00800.1	12	22702307	NC_012018.3 (14705472..14706742	Vitis vinifera
VIT_215s0024g00040.1	18	29360087	NC_012024.3 (29067609..29070184	Vitis vinifera
VIT_207s0031g01530.1	8	22385789	NC_012014.3 (14979242..14980109	Vitis vinifera
VIT_208s0007g00780.1	12	22702307	NC_012018.3 (9272172..9273893)	Vitis vinifera
VIT_208s0007g02190.1	7	21026613	NC_012013.3 (20057758..20077397)	Vitis vinifera
VIT_211s0016g00730.1	7	21026613	NC_012013.3 (4604973..4605929	Vitis vinifera
VIT_212s0028g00320.1	15	20304914	NC_012021.3 (124043..125348)	Vitis vinifera
VIT_212s0057g00630.1	11	19818926	NC_012017.3 (700246..700564)	Vitis vinifera
VIT_212s0055g01110.1	10	18140952	NC_012016.3 (5026472..5027476)	Vitis vinifera
VIT_213s0019g04140.1	13	24396255	NC_012019.3 (5428773..5430347)	Vitis vinifera
VIT_216s0039g02710.1	16	22053297	NC_012022.3 (3083375..3084740	Vitis vinifera
VIT_217s0000g06350.1	11	19818926	NC_012017.3 (701246..703564)	Vitis vinifera
VIT_218s0001g10550.1	4	23867706	NC_012010.3 (690467..694916)	Vitis vinifera
VIT_218s0001g02740.1	8	22385789	NC_012014.3 (16224930..16226090)	Vitis vinifera
VIT_218s0089g01170.1	7	21026613	NC_012013.3 (17627128..17630179)	Vitis vinifera
VIT_219s0014g03660.1	12	22702307	NC_012018.3 (931602..932647	Vitis vinifera
VIT_219s0014g00160.1	19	24021853	NC_012025.3 (3777321..3779253	Vitis vinifera
VIT_210s0003g02900.1	18	29360087	NC_012024.3 (8871554..8873818)	Vitis vinifera
VIT_207s0005g02220.1	17	17126926	NC_012023.3 (6886211..6887519	Vitis vinifera
VIT_207s0031g03200.1	3	19341862	NC_012009.3 (6033720..6036626	Vitis vinifera

VIT_210s0003g02890.1	19	24021853	NC_012025.3 (150445..151569	Vitis vinifera
VIT_218s0072g00120.1	18	29360087	NC_012024.3 (2905749..2907431)	Vitis vinifera
VIT_218s0001g06720.1	10	18140952	NC_012016.3 (5014519..5015467	Vitis vinifera
VIT_200s0181g00180.1	18	29360087	NC_012024.3 (19152960..19156535)	Vitis vinifera
VIT_200s0181g00200.1		1790842	NW_003724219.1(179263..182546)	Vitis vinifera
VIT_200s0204g00170.1	18	29360087	NC_012024.3 (28067607..28070184	Vitis vinifera
VIT_205s0020g04110.2		1790842	NW_003724191.1(1658055..1662187)	Vitis vinifera
VIT_201s0010g03620.1	1	23037639	NC_012007.3 (20954786..20957045	Vitis vinifera
VIT_218s0122g00430.1	10	18140952	NC_012016.3 (5015472..5016476)	Vitis vinifera
VIT_218s0122g00430.1	18	29360087	NC_012024.3 (380111..387381	Vitis vinifera
VOLCADRAFT_109142		5981844	NW_003307545.1(4023885..4058642)	Volvox carteri
VOLCADRAFT_83375		5981844	NW_003307545.1(4430423..4432081)	Volvox carteri
VOLCADRAFT_100204		2777111	NW_003307547.1(2286150..2286941)	Volvox carteri
VOLCADRAFT_80312		2401260	NW_003307550.1(678299..730893)	Volvox carteri
VOLCADRAFT_105072		2412439	NW_003307551.1(763412..76532)	Volvox carteri
VOLCADRAFT_109843		2307048	NW_003307552.1(506845..532212)	Volvox carteri
VOLCADRAFT_57605		2108072	NW_003307555.1(1327231..1329832)	Volvox carteri
VOLCADRAFT_77072		2038975	NW_003307561.1(86158..111738)	Volvox carteri
VOLCADRAFT_110044		1800600	NW_003307566.1(696400..695000)	Volvox carteri
VOLCADRAFT_81918		1657398	NW_003307567.1(1394200..139234)	Volvox carteri
VOLCADRAFT_81675		1648468	NW_003307568.1(150079..193655)	Volvox carteri
VOLCADRAFT_109135		1644120	NW_003307569.1(1196423..1198625)	Volvox carteri
VOLCADRAFT_80470		1555513	NW_003307574.1(196262..198158)	Volvox carteri
VOLCADRAFT_45832		1555513	NW_003307574.1(297600..300421)	Volvox carteri
VOLCADRAFT_108856		1555513	NW_003307574.1(41500..416589)	Volvox carteri
VOLCADRAFT_87935		1267045	NW_003307582.1(263618..321560)	Volvox carteri
VOLCADRAFT_77575		954740	NW_003307597.1(560820..591671)	Volvox carteri
VOLCADRAFT_104631		968918	NW_003307598.1(149925..169552)	Volvox carteri
VOLCADRAFT_108852		853771	NW_003307603.1(204342..211456)	Volvox carteri
VOLCADRAFT_106964		753185	NW_003307611.1(78126..82351)	Volvox carteri
VOLCADRAFT_64158		648959	NW_003307616.1(307949..340029)	Volvox carteri
VOLCADRAFT_80066		312566	NW_003307641.1(184014..205678)	Volvox carteri
VOLCADRAFT_108288		312566	NW_003307641.1(276459..307434)	Volvox carteri
VOLCADRAFT_85008		298891	NW_003307646.1(155655.203576)	Volvox carteri
VOLCADRAFT_108699		298891	NW_003307646.1(252156..272630)	Volvox carteri
VOLCADRAFT_78398		298891	NW_003307646.1(256200..272630)	Volvox carteri
VOLCADRAFT_108301		258463	NW_003307651.1(150079..193655)	Volvox carteri
VOLCADRAFT_78096		8276	NW_003307851.1(228..2525)	Volvox carteri
VOLCADRAFT_77985		8725	NW_003307959.1(7569..14359)	Volvox carteri
VOLCADRAFT_84524		6942	NW_003308092.1(144..1063)	Volvox carteri
GRMZM2G131489	4	246994605	NC_024462.2 (241059728..241060959	Zea mays
GRMZM2G153184	1	308452471	NC_050096.1 (218541179..218542622	Zea mays
GRMZM2G414192	1	308452471	NC_050096.1 (253786679..253787914	Zea mays
GRMZM2G127512	1	308452471	NC_050096.1 (253819035..253820312)	Zea mays

GRMZM2G047095	2	243675191	NC_050097.1 (177729739..177737314)	Zea mays
GRMZM2G117412	2	243675191	NC_050097.1 (189051396..189053773)	Zea mays
GRMZM2G117412	2	243675191	NC_050097.1 (211713147..211714533	Zea mays
GRMZM2G038519	2	243675191	NC_050097.1 (213803814..213810515)	Zea mays
GRMZM2G149428	2	243675191	NC_050097.1 (226115138..226117234	Zea mays
GRMZM2G039996	2	243675191	NC_050097.1 (2292282..2293458	Zea mays
GRMZM2G355752	2	243675191	NC_050097.1 (239759753..239760662)	Zea mays
GRMZM2G154752	3	238017767	NC_050098.1 (159820342..159822736	Zea mays
GRMZM2G077333	3	238017767	NC_050098.1 (179564393..179566837	Zea mays
GRMZM2G019807	3	238017767	NC_050098.1 (227250926..227252109)	Zea mays
GRMZM2G120619	3	238017767	NC_050098.1 (228968484..228969525)	Zea mays
GRMZM2G104549	3	238017767	NC_050098.1 (229020920..229021944)	Zea mays
GRMZM2G036582	3	238017767	NC_050098.1 (229059342..229060422)	Zea mays
GRMZM2G004305	3	238017767	NC_050098.1 (229147830..229149285)	Zea mays
GRMZM2G331860	4	250330460	NC_050099.1 (12193125..12195063)	Zea mays
GRMZM2G104405	4	250330460	NC_050099.1 (244395879..244397110	Zea mays
GRMZM2G204386	4	250330460	NC_050099.1 (2474666354..247457813)	Zea mays
GRMZM2G036880	4	250330460	NC_050099.1 (247467354..247468813)	Zea mays
GRMZM2G234785	4	250330460	NC_050099.1 (86380150..86381372)	Zea mays
GRMZM2G216490	5	226353449	NC_050100.1 (177692071..177698665)	Zea mays
GRMZM2G154380	5	226353449	NC_050100.1 (20116113..20179229	Zea mays
GRMZM2G103101	5	226353449	NC_050100.1 (218240084..218241745	Zea mays
GRMZM2G159013	5	226353449	NC_050100.1 (69688954..69709900	Zea mays
GRMZM2G477236	5	226353449	NC_050100.1 (75151291..75152886)	Zea mays
GRMZM2G092311	5	226353449	NC_050100.1 (87413366..87414477)	Zea mays
GRMZM2G006380	6	181357234	NC_050101.1 (176348426..176349478)	Zea mays
GRMZM2G157030	7	185808916	NC_050102.1 (153471519..153472596	Zea mays
GRMZM2G436210	7	185808916	NC_050102.1 (166291674..166293098	Zea mays
GRMZM2G214359	7	185808916	NC_050102.1 (166624385..166625598)	Zea mays
GRMZM2G072280	7	185808916	NC_050102.1 (169327208..169328863)	Zea mays
GRMZM2G012324	7	185808916	NC_050102.1 (5294557..5295557	Zea mays
GRMZM2G133920	7	185808916	NC_050102.1 (86258636..86270670)	Zea mays
GRMZM2G206308	7	185808916	NC_050102.1 (9861703..9862580)	Zea mays
GRMZM2G146870	8	182411202	NC_050103.1 (144224338..144225496)	Zea mays
GRMZM2G004305	8	182411202	NC_050103.1 (145361964..145363001)	Zea mays
GRMZM2G155216	8	182411202	NC_050103.1 (72078494..72079504	Zea mays
GRMZM2G010884	9	163004744	NC_050104.1 (107818198..107819512	Zea mays
GRMZM2G110284	10	152435371	NC_050105.1 (146850321..146851577)	Zea mays
GRMZM2G092427	10	152435371	NC_050105.1 (150168727..150169927)	Zea mays
ZOSMA_87G00080		615966	scaffold_10(346571..345942)	Zostera marina
ZOSMA_60G00630		756304	scaffold_101(229163..230220)	Zostera marina
ZOSMA_48G00340		810575	scaffold_105(487460..488680)	Zostera marina
ZOSMA_361G00150		148145	scaffold_106(425657..426517)	Zostera marina
ZOSMA_34G00670		918970	scaffold_121(77935..77111)	Zostera marina

ZOSMA_31G00600	933748	scaffold_122(272995..272420)	Zostera marina
ZOSMA_25G00660	933748	scaffold_122(275498..276253)	Zostera marina
ZOSMA_172G00410	933748	scaffold_122(280097..280342)	Zostera marina
ZOSMA_152G00070	933748	scaffold_122(286488..285613)	Zostera marina
ZOSMA_106G00570	858898	scaffold_137(404270..404596)	Zostera marina
ZOSMA_105G00660	858898	scaffold_137(551291..550998)	Zostera marina
ZOSMA_101G00320	858898	scaffold_137(790571..791056)	Zostera marina
ZOSMA_89G01210	634645	scaffold_89(165865..166512)	Zostera marina
ZOSMA_83G00260	647443	scaffold_83(104509..105244)	Zostera marina
ZOSMA_82G00850	664480	scaffold_82(160616..160371)	Zostera marina
ZOSMA_655G00010	749424	scaffold_655(684299..683544)	Zostera marina
ZOSMA_59G00610	724359	scaffold_59(23655..27462)	Zostera marina
ZOSMA_31G00760	933748	scaffold_31(181222..180908)	Zostera marina
ZOSMA_278G00220	322635	scaffold_278(236459..235836)	Zostera marina
ZOSMA_25G01660	1009911	scaffold_25(684299..683544)	Zostera marina
ZOSMA_217G00280	1009911	scaffold_25(240664..240840）	Zostera marina
ZOSMA_186G00090	1009911	scaffold_25(474117..474846)	Zostera marina
ZOSMA_173G00090	322635	scaffold_278(236471..235803)	Zostera marina
ZOSMA_152G00140	933748	scaffold_31(280030..280954)	Zostera marina
ZOSMA_152G00200	530396	scaffold_34(517138..523402)	Zostera marina
ZOSMA_151G00070	424709	scaffold_151(89126..90898)	Zostera marina
ZOSMA_137G00210	466922	scaffold_48(21292..22227)	Zostera marina
ZOSMA_137G00310	969424	scaffold_59(22432..23046)	Zostera marina
ZOSMA_137G00320	969424	scaffold_59(953957..953160)	Zostera marina
ZOSMA_122G00160	764369	scaffold_60(720674..722163)	Zostera marina
ZOSMA_122G00170	724339	scaffold_655(684299..683898)	Zostera marina
ZOSMA_122G00210	694169	scaffold_81(579251..581304)	Zostera marina
ZOSMA_122G00230	314379	scaffold_82(224997..225695)	Zostera marina
ZOSMA_121G00630	664469	scaffold_83(579651..580304)	Zostera marina
ZOSMA_10G00190	534319	scaffold_87(46001..46354)	Zostera marina
ZOSMA_82G00410	464329	scaffold_89(374866..374232)	Zostera marina

Table S2. Conserved motif sequence of all obtained LHC genes from 42 species.

Name	Motif sequence
Motif1	YVGMRYWHPFTEEAIEQIKRDGITKLVVLPLYPQFSISTSGSSLRLLENI
Motif2	YQSRVGPVEWLKPYTDETIVELGQKGVKSLLAVPISFVSEHIETLEEIDV
Motif3	AEIWNGRFAMLGLVA
Motif4	EVMIFFSAHGVPLAYVEEAGDPYKAEMEECVDLIMEELEKR
Motif5	FREDEYLVNMQHTVIPSWYQREGYIKAMADLIEKELEKFDN
Motif6	HSHVGEEKVGVLLLNLGGPETLNDVQPFLFNLFAD
Motif7	PDIIRLPRLFRFLQKPLAQFISVLRAPKSKEGYASIGGGSP
Motif8	EYKELALESGIENWGRVPALGCEPTFISDLADAVIESLPYV
Motif9	NLEARQSLVPLGSVEELLATYDSQRRELPPPVTVWEWGWTK
Motif10	KASTSIWDALAFSGPAPERINGRLAMVGF
Motif11	AYLVDSLTGVGLVDQMGNFFC

Motif12	TLLFVAVVGVLLIRKNEDLDNIKKLIEETTFYDKQWQATWQ
Motif13	NPESSSNDDPVVFDTSIIPWWAWMKRFHL
Motif14	SIIGEIITGKGALAQLNIETGVPINEIEPLVLFNVVFFFIAAINPGTGKF
Motif15	WFAYTVAVLSVASLVPLLQGESAE
Motif16	AGLSADPETFKRYRELELIHARWAMLGAL
Motif17	LRKITDEQADALKMALEEKNV
Motif18	GKVDWDAVIDAEVVRRKWLED
Motif19	IKFEDARWVNGTWDLKQFEKD
Motif20	VWFKAGAQIFQEGGLDYLGNPSLVHAQNIVATLACQVILMGLVEGYRVNG
Motif21	MAMSSFAGAAVLPRGSAGRFGARSLPALGRRALVIRAQTEGPSAPPPNKP
Motif22	DDKNVITLEFQRQKAKELQEYFKQKKLE
Motif23	IPIYEAEPLLLFFILFTLLGAIGALGDRG
Motif24	KVKDTDSDMDMMHYLTKMFFGSVLAFFLLLSPRLVSAFRNT

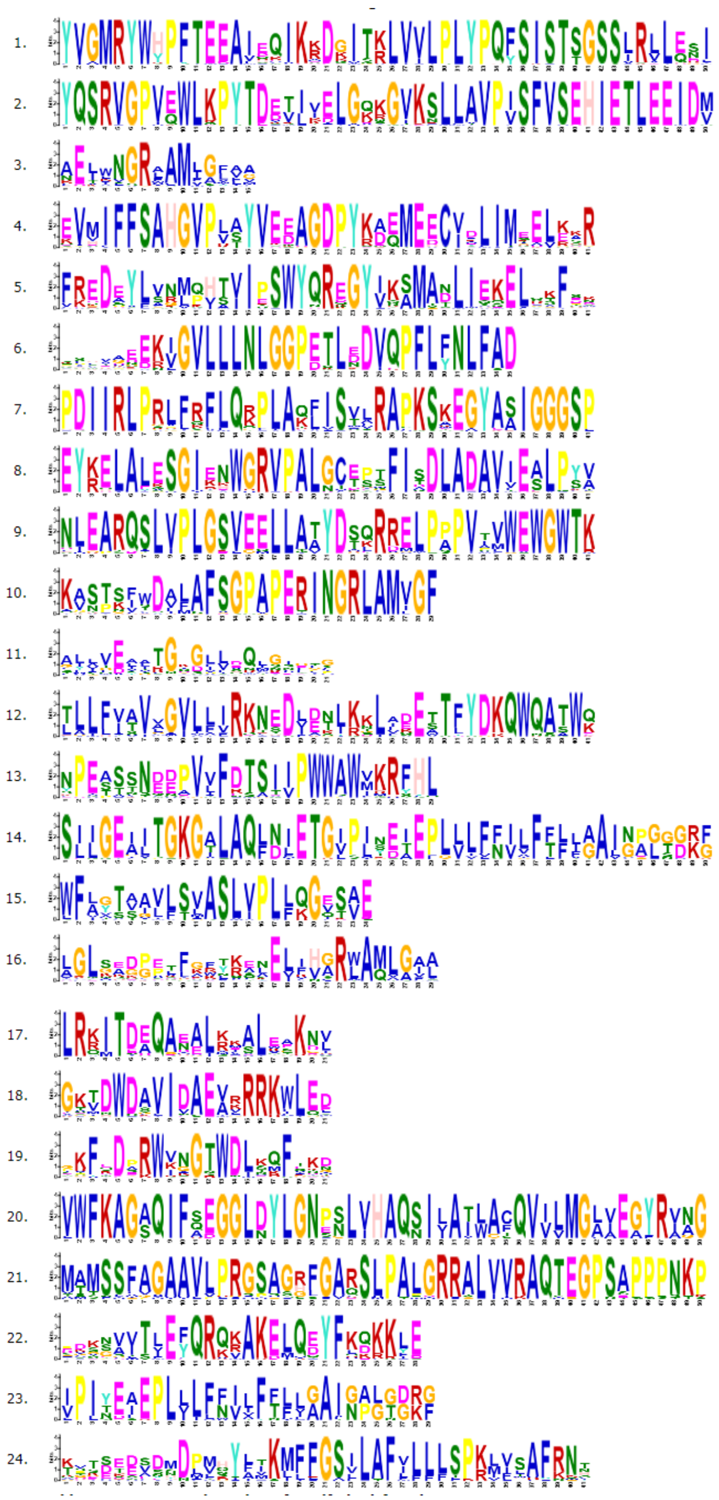

Figure S1. Motifs of the proteins.

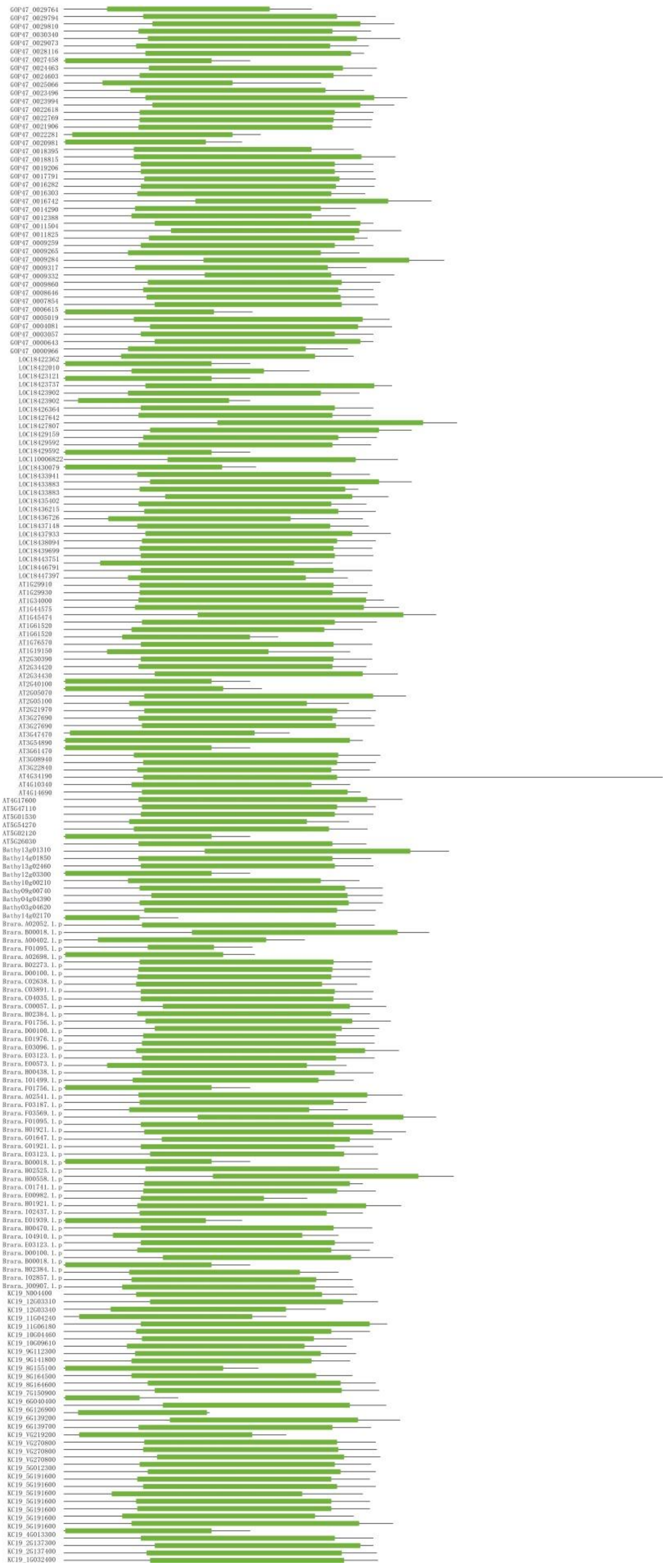
GOP47_0029764
GOP47_0029794
GOP47_0029810
GOP47_0030340
GOP47_0029073
GOP47_0028116
GOP47_0027458
GOP47_0024463
GOP47_0024603
GOP47_0025066
GOP47_0023496
GOP47_0023994
GOP47_0022618
GOP47_0022769
GOP47_0021906
GOP47_0022281
GOP47_0020981
GOP47_0018395
GOP47_0018815
GOP47_0019206
GOP47_0017791
GOP47_0016282
GOP47_0016303
GOP47_0016742
GOP47_0014290
GOP47_0012388
GOP47_0011504
GOP47_0011825
GOP47_0009259
GOP47_0009265
GOP47_0009284
GOP47_0009317
GOP47_0009332
GOP47_0009860
GOP47_0008646
GOP47_0007854
GOP47_0006615
GOP47_0005019
GOP47_0004081
GOP47_0003057
GOP47_0000643
GOP47_0000966
LOC18422362
LOC18422010
LOC18423121
LOC18423737
LOC18423902
LOC18423902
LOC18426364
LOC18427642
LOC18427807
LOC18429159
LOC18429592
LOC18429592
LOC110006822
LOC18430079
LOC18433941
LOC18433883
LOC18433883
LOC18435402
LOC18436215
LOC18436726
LOC18437148
LOC18437933
LOC18438094
LOC18439699
LOC18443751
LOC18446791
LOC18447397
AT1G29910
AT1G29930
AT1G34000
AT1G44575
AT1G45474
AT1G61520
AT1G61520
AT1G76570
AT1G19150
AT2G30390
AT2G34420
AT2G34430
AT2G40100
AT2G05070
AT2G05100
AT2G21970
AT3G27690
AT3G27690
AT3G47470
AT3G54890
AT3G61470
AT3G08940
AT3G22840
AT4G34190
AT4G10340
AT4G14690
AT4G17600
AT5G47110
AT5G01530
AT5G54270
AT5G02120
AT5G26030
Bathy13g01310
Bathy14g01850
Bathy13g02460
Bathy12g03300
Bathy10g00210
Bathy09g00740
Bathy04g04390
Bathy03g04620
Bathy14g02170
Brara.A02052.1.p
Brara.B00018.1.p
Brara.A00402.1.p
Brara.F01095.1.p
Brara.A02698.1.p
Brara.B02273.1.p
Brara.D00100.1.p
Brara.C02638.1.p
Brara.C03891.1.p
Brara.C04035.1.p
Brara.C00057.1.p
Brara.H02384.1.p
Brara.F01756.1.p
Brara.D00100.1.p
Brara.E01976.1.p
Brara.E03096.1.p
Brara.E03123.1.p
Brara.E00573.1.p
Brara.H00438.1.p
Brara.I01499.1.p
Brara.F01756.1.p
Brara.A02541.1.p
Brara.F03187.1.p
Brara.F03569.1.p
Brara.F01095.1.p
Brara.H01921.1.p
Brara.G01647.1.p
Brara.G01921.1.p
Brara.E03123.1.p
Brara.B00018.1.p
Brara.H02525.1.p
Brara.H00558.1.p
Brara.C01741.1.p
Brara.E00982.1.p
Brara.H01921.1.p
Brara.I02437.1.p
Brara.E01939.1.p
Brara.H00470.1.p
Brara.I04910.1.p
Brara.E03123.1.p
Brara.D00100.1.p
Brara.B00018.1.p
Brara.H02384.1.p
Brara.I02857.1.p
Brara.J00907.1.p
KC19_N004400
KC19_12G03310
KC19_12G03340
KC19_11G04240
KC19_11G06180
KC19_10G04460
KC19_10G09610
KC19_9G112300
KC19_9G141800
KC19_8G155100
KC19_8G164500
KC19_8G164600
KC19_7G150900
KC19_6G040400
KC19_6G126900
KC19_6G139200
KC19_6G139700
KC19_VG219200
KC19_VG270800
KC19_VG270800
KC19_VG270800
KC19_5G012300
KC19_5G191600
KC19_5G191600
KC19_5G191600
KC19_5G191600
KC19_5G191600
KC19_5G191600
KC19_5G191600
KC19_4G013300
KC19_2G137300
KC19_2G137400
KC19_1G032400

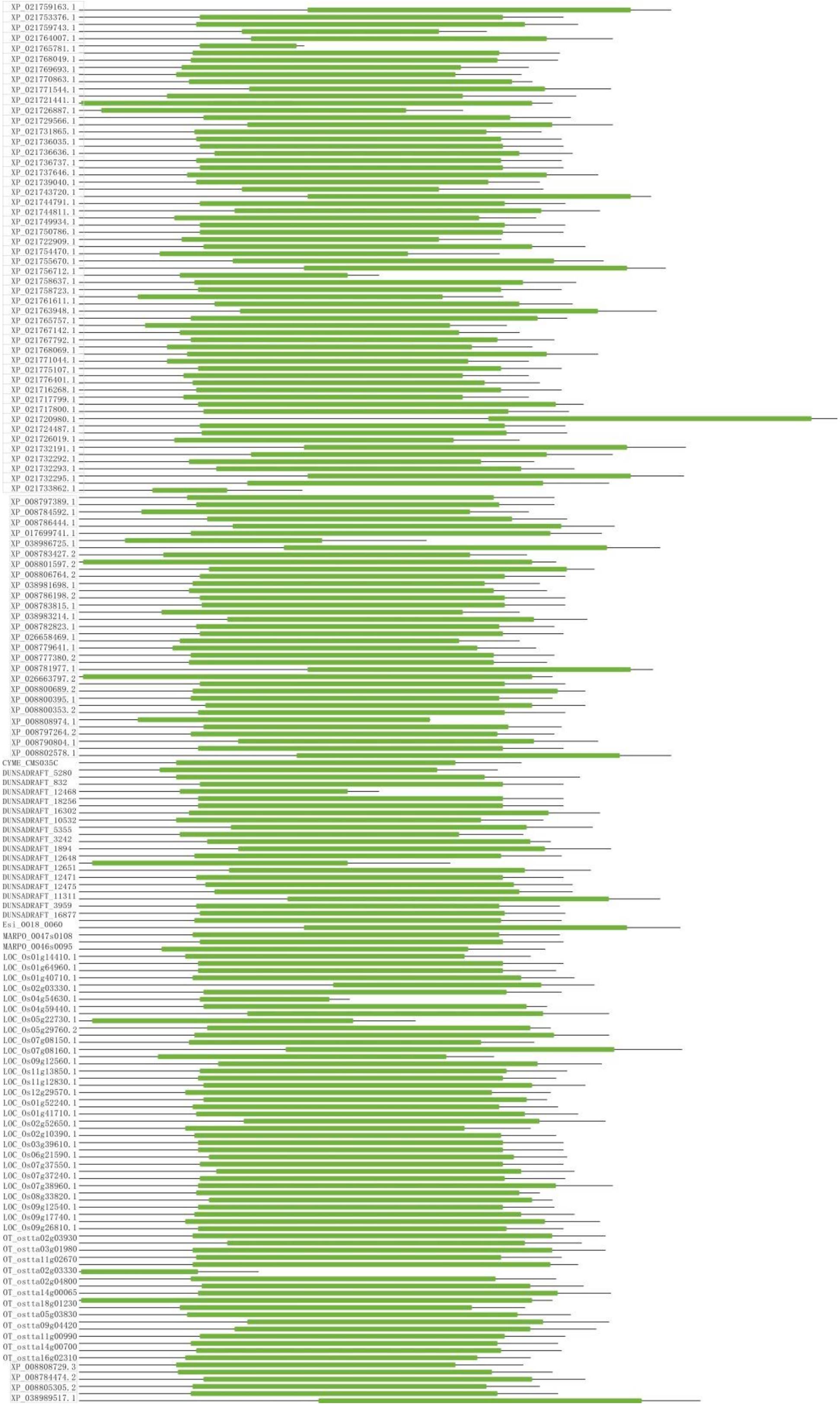

XP_021759163.1
XP_021753376.1
XP_021759743.1
XP_021764007.1
XP_021765781.1
XP_021768049.1
XP_021769693.1
XP_021770863.1
XP_021771544.1
XP_021721441.1
XP_021726887.1
XP_021729566.1
XP_021731865.1
XP_021736035.1
XP_021736636.1
XP_021736737.1
XP_021737646.1
XP_021739040.1
XP_021743720.1
XP_021744791.1
XP_021744811.1
XP_021749934.1
XP_021750786.1
XP_021722909.1
XP_021754470.1
XP_021755670.1
XP_021756712.1
XP_021758637.1
XP_021758723.1
XP_021761611.1
XP_021763948.1
XP_021765757.1
XP_021767142.1
XP_021767792.1
XP_021768069.1
XP_021771044.1
XP_021775107.1
XP_021776401.1
XP_021716268.1
XP_021717799.1
XP_021717800.1
XP_021720980.1
XP_021724487.1
XP_021726019.1
XP_021732191.1
XP_021732292.1
XP_021732293.1
XP_021732295.1
XP_021733862.1
XP_008797389.1
XP_008784592.1
XP_008786444.1
XP_017699741.1
XP_038986725.1
XP_008783427.2
XP_008801597.2
XP_008806764.2
XP_038981698.1
XP_008786198.2
XP_008783815.1
XP_038983214.1
XP_008782823.1
XP_026658469.1
XP_008779641.1
XP_008777380.2
XP_008781977.1
XP_026663797.2
XP_008800689.2
XP_008800395.1
XP_008800353.2
XP_008808974.1
XP_008797264.2
XP_008790804.1
XP_008802578.1
CYME_CMS035C
DUNSADRAFT_5280
DUNSADRAFT_832
DUNSADRAFT_12468
DUNSADRAFT_18256
DUNSADRAFT_16302
DUNSADRAFT_10532
DUNSADRAFT_5355
DUNSADRAFT_3242
DUNSADRAFT_1894
DUNSADRAFT_12648
DUNSADRAFT_12651
DUNSADRAFT_12471
DUNSADRAFT_12475
DUNSADRAFT_11311
DUNSADRAFT_3959
DUNSADRAFT_16877
Esi_0018_0060
MARPO_0047s0108
MARPO_0046s0095
LOC_Os01g14410.1
LOC_Os01g64960.1
LOC_Os01g40710.1
LOC_Os02g03330.1
LOC_Os04g54630.1
LOC_Os04g59440.1
LOC_Os05g22730.1
LOC_Os05g29760.2
LOC_Os07g08150.1
LOC_Os07g08160.1
LOC_Os09g12560.1
LOC_Os11g13850.1
LOC_Os11g12830.1
LOC_Os12g29570.1
LOC_Os01g52240.1
LOC_Os01g41710.1
LOC_Os02g52650.1
LOC_Os02g10390.1
LOC_Os03g39610.1
LOC_Os06g21590.1
LOC_Os07g37550.1
LOC_Os07g37240.1
LOC_Os07g38960.1
LOC_Os08g33820.1
LOC_Os09g12540.1
LOC_Os09g17740.1
LOC_Os09g26810.1
OT_ostta02g03930
OT_ostta03g01980
OT_ostta11g02670
OT_ostta02g03330
OT_ostta02g04800
OT_ostta14g00065
OT_ostta18g01230
OT_ostta05g03830
OT_ostta09g04420
OT_ostta11g00990
OT_ostta14g00700
OT_ostta16g02310
XP_008808729.3
XP_008784474.2
XP_008805305.2
XP_038989517.1

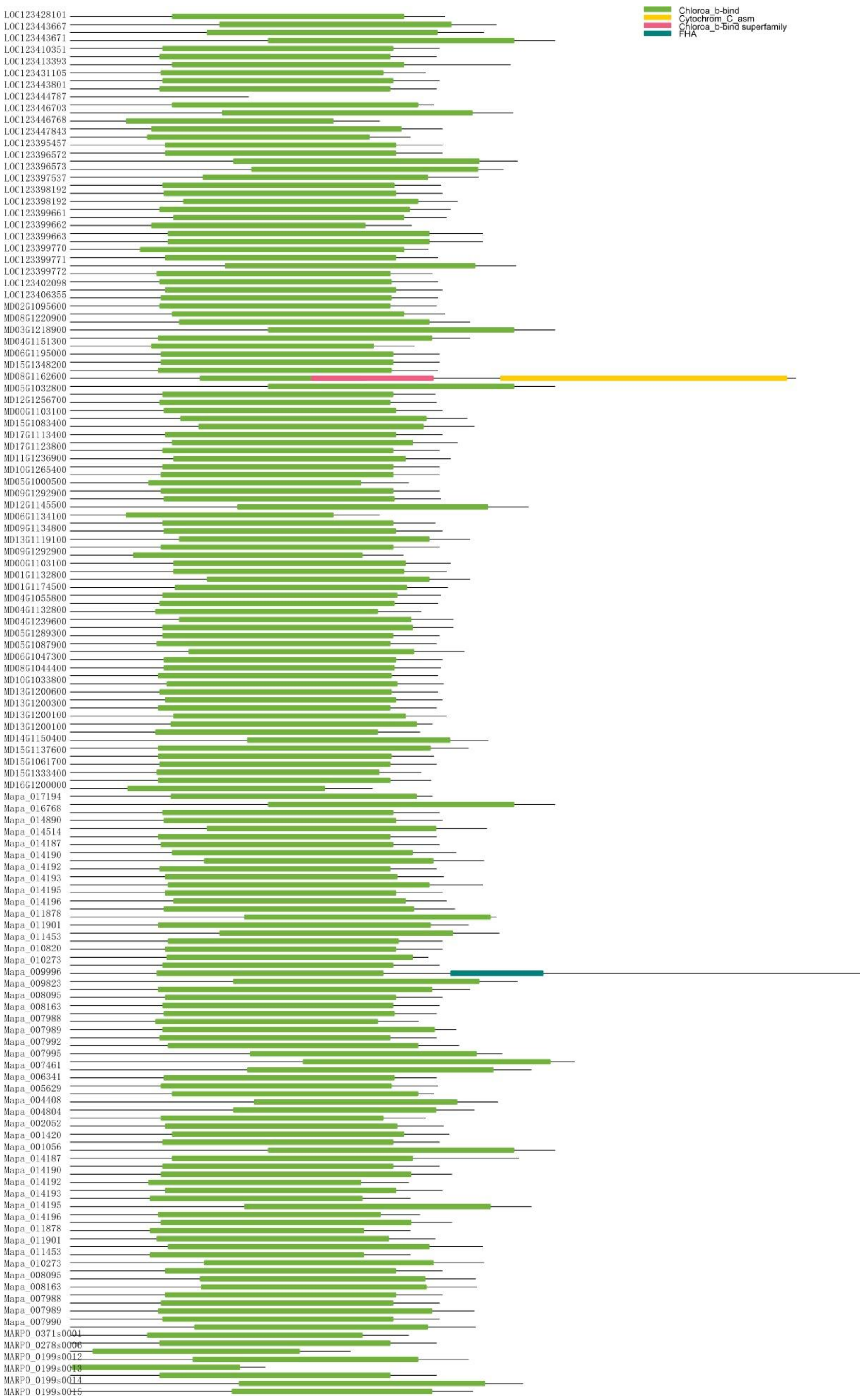

Chloroa_b-bind
Cytochrom_C_asm
Chloroa_b-bind superfamily
FHA
LOC123428101
LOC123443667
LOC123443671
LOC123410351
LOC123413393
LOC123431105
LOC123443801
LOC123444787
LOC123446703
LOC123446768
LOC123447843
LOC123395457
LOC123396572
LOC123396573
LOC123397537
LOC123398192
LOC123398192
LOC123399661
LOC123399662
LOC123399663
LOC123399770
LOC123399771
LOC123399772
LOC123402098
LOC123406355
MD02G1095600
MD08G1220900
MD03G1218900
MD04G1151300
MD06G1195000
MD15G1348200
MD08G1162600
MD05G1032800
MD12G1256700
MD00G1103100
MD15G1083400
MD17G1113400
MD17G1123800
MD11G1236900
MD10G1265400
MD05G1000500
MD09G1292900
MD12G1145500
MD06G1134100
MD09G1134800
MD13G1119100
MD09G1292900
MD00G1103100
MD01G1132800
MD01G1174500
MD04G1055800
MD04G1132800
MD04G1239600
MD05G1289300
MD05G1087900
MD06G1047300
MD08G1044400
MD10G1033800
MD13G1200600
MD13G1200300
MD13G1200100
MD13G1200100
MD14G1150400
MD15G1137600
MD15G1061700
MD15G1333400
MD16G1200000
Mapa_017194
Mapa_016768
Mapa_014890
Mapa_014514
Mapa_014187
Mapa_014190
Mapa_014192
Mapa_014193
Mapa_014195
Mapa_014196
Mapa_011878
Mapa_011901
Mapa_011453
Mapa_010820
Mapa_010273
Mapa_009996
Mapa_009823
Mapa_008095
Mapa_008163
Mapa_007988
Mapa_007989
Mapa_007992
Mapa_007995
Mapa_007461
Mapa_006341
Mapa_005629
Mapa_004408
Mapa_004804
Mapa_002052
Mapa_001420
Mapa_001056
Mapa_014187
Mapa_014190
Mapa_014192
Mapa_014193
Mapa_014195
Mapa_014196
Mapa_011878
Mapa_011901
Mapa_011453
Mapa_010273
Mapa_008095
Mapa_008163
Mapa_007988
Mapa_007989
Mapa_007990
MARPO_0371s0001
MARPO_0278s0006
MARPO_0199s0012
MARPO_0199s0013
MARPO_0199s0014
MARPO_0199s0015

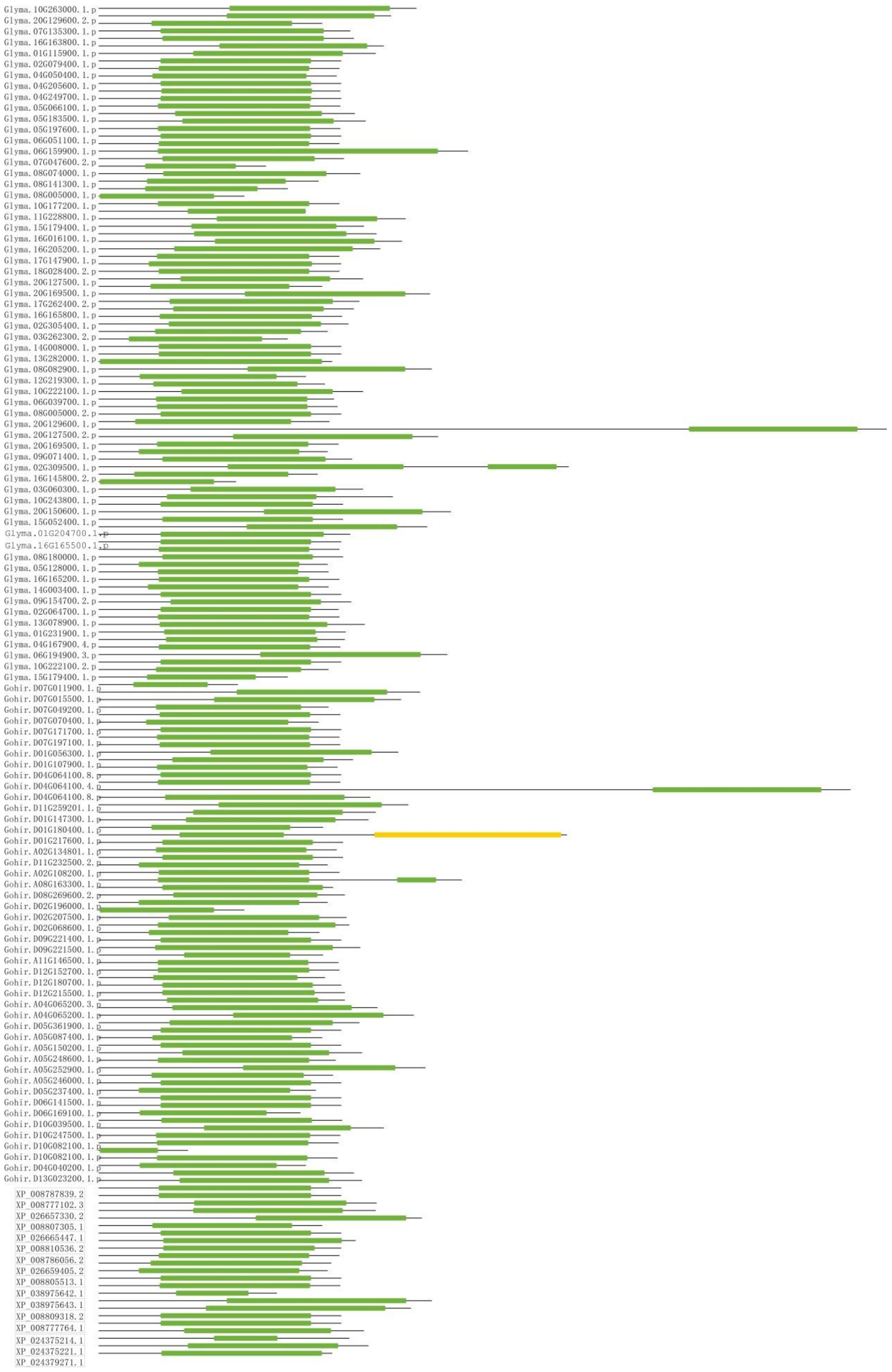
Glyma.10G263000.1.p
Glyma.20G129600.2.p
Glyma.07G135300.1.p
Glyma.16G163800.1.p
Glyma.01G115900.1.p
Glyma.02G079400.1.p
Glyma.04G050400.1.p
Glyma.04G205600.1.p
Glyma.04G249700.1.p
Glyma.05G066100.1.p
Glyma.05G183500.1.p
Glyma.05G197600.1.p
Glyma.06G051100.1.p
Glyma.06G159900.1.p
Glyma.07G047600.2.p
Glyma.08G074000.1.p
Glyma.08G141300.1.p
Glyma.08G005000.1.p
Glyma.10G177200.1.p
Glyma.11G228800.1.p
Glyma.15G179400.1.p
Glyma.16G016100.1.p
Glyma.16G205200.1.p
Glyma.17G147900.1.p
Glyma.18G028400.2.p
Glyma.20G127500.1.p
Glyma.20G169500.1.p
Glyma.17G262400.2.p
Glyma.16G165800.1.p
Glyma.02G305400.1.p
Glyma.03G262300.2.p
Glyma.14G008000.1.p
Glyma.13G282000.1.p
Glyma.08G082900.1.p
Glyma.12G219300.1.p
Glyma.10G222100.1.p
Glyma.06G039700.1.p
Glyma.08G005000.2.p
Glyma.20G129600.1.p
Glyma.20G127500.2.p
Glyma.20G169500.1.p
Glyma.09G071400.1.p
Glyma.02G309500.1.p
Glyma.16G145800.2.p
Glyma.03G060300.1.p
Glyma.10G243800.1.p
Glyma.20G150600.1.p
Glyma.15G052400.1.p
Glyma.01G204700.1.p
Glyma.16G165500.1.p
Glyma.08G180000.1.p
Glyma.05G128000.1.p
Glyma.16G165200.1.p
Glyma.14G003400.1.p
Glyma.09G154700.2.p
Glyma.02G064700.1.p
Glyma.13G078900.1.p
Glyma.01G231900.1.p
Glyma.04G167900.4.p
Glyma.06G194900.3.p
Glyma.10G222100.2.p
Glyma.15G179400.1.p
Gohir.D07G011900.1.p
Gohir.D07G015500.1.p
Gohir.D07G049200.1.p
Gohir.D07G070400.1.p
Gohir.D07G171700.1.p
Gohir.D07G197100.1.p
Gohir.D01G056300.1.p
Gohir.D01G107900.1.p
Gohir.D04G064100.8.p
Gohir.D04G064100.4.p
Gohir.D04G064100.8.p
Gohir.D11G259201.1.p
Gohir.D01G147300.1.p
Gohir.D01G180400.1.p
Gohir.D01G217600.1.p
Gohir.A02G134801.1.p
Gohir.D11G232500.2.p
Gohir.A02G108200.1.p
Gohir.A08G163300.1.p
Gohir.D08G269600.2.p
Gohir.D02G196000.1.p
Gohir.D02G207500.1.p
Gohir.D02G068600.1.p
Gohir.D09G221400.1.p
Gohir.D09G221500.1.p
Gohir.A11G146500.1.p
Gohir.D12G152700.1.p
Gohir.D12G180700.1.p
Gohir.D12G215500.1.p
Gohir.A04G065200.3.p
Gohir.A04G065200.1.p
Gohir.D05G361900.1.p
Gohir.A05G087400.1.p
Gohir.A05G150200.1.p
Gohir.A05G248600.1.p
Gohir.A05G252900.1.p
Gohir.A05G246000.1.p
Gohir.D05G237400.1.p
Gohir.D06G141500.1.p
Gohir.D06G169100.1.p
Gohir.D10G039500.1.p
Gohir.D10G247500.1.p
Gohir.D10G082100.1.p
Gohir.D10G082100.1.p
Gohir.D04G040200.1.p
Gohir.D13G023200.1.p
XP_008787839.2
XP_008777102.3
XP_026657330.2
XP_008807305.1
XP_026665447.1
XP_008810536.2
XP_008786056.2
XP_026659405.2
XP_008805513.1
XP_038975642.1
XP_038975643.1
XP_008809318.2
XP_008777764.1
XP_024375214.1
XP_024375221.1
XP_024379271.1

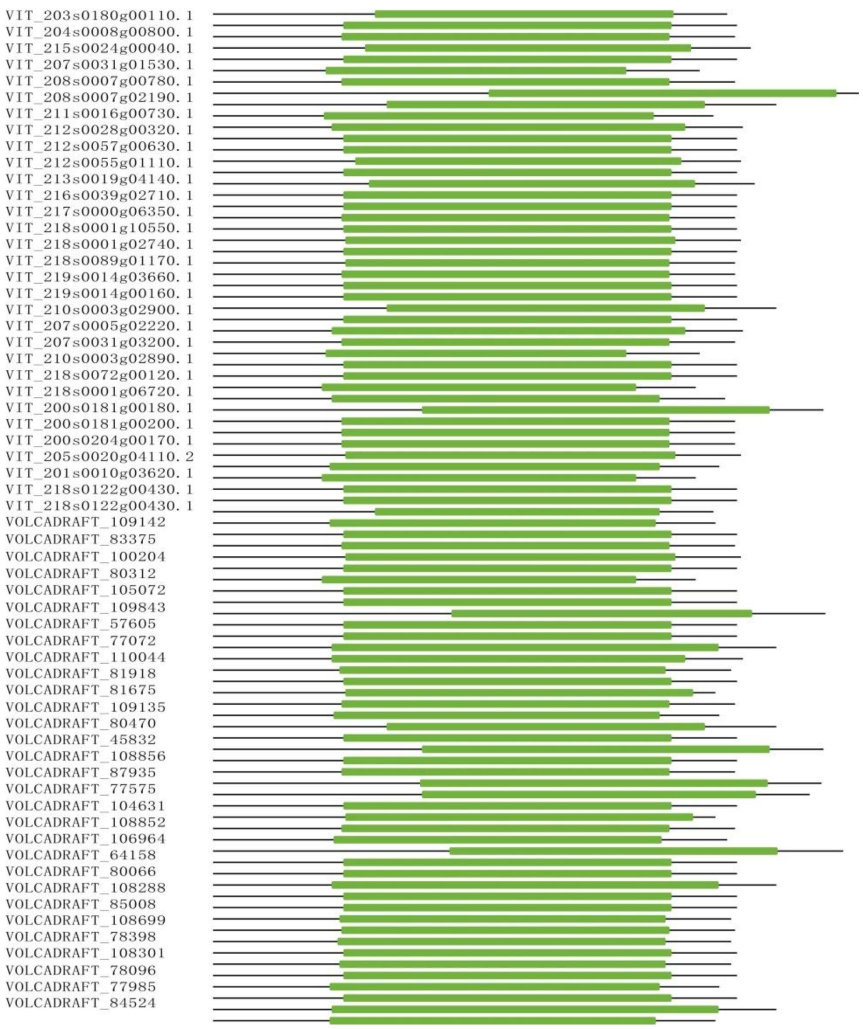

Figure S2. Domains of the Lhc proteins
